# Ginger nanoparticles mediated induction of Foxa2 prevents high-fat diet-induced insulin resistance

**DOI:** 10.7150/thno.62514

**Published:** 2022-01-01

**Authors:** Anil Kumar, Kumaran Sundaram, Yun Teng, Jingyao Mu, Mukesh K Sriwastva, Lifeng Zhang, Joshua L. Hood, Jun Yan, Xiang Zhang, Juw Won Park, Michael L Merchant, Huang-Ge Zhang

**Affiliations:** 1 James Graham Brown Cancer Center, Department of Microbiology & Immunology, University of Louisville, KY 40202, USA.; 2 Department of Pharmacology and Toxicology, University of Louisville, Louisville, KY40202, USA.; 3 Department of Computer Engineering and Computer Science, University of Louisville, KY40202, USA.; 4 KBRIN Bioinformatics Core, University of Louisville, Louisville, KY 40202, USA.; 5 Kidney Disease Program and Clinical Proteomics Center, University of Louisville, Louisville, KY, USA.; 6 Robley Rex Veterans Affairs Medical Center, Louisville, KY 40206, USA.

**Keywords:** Ginger-derived nanoparticles, phosphatidic acid, Foxa2, insulin resistance, exosomes, skin inflammation, lifespan

## Abstract

**Rationale:** The obesity epidemic has expanded globally, due in large part to the increased consumption of high-fat diets (HFD), and has increased the risk of major chronic diseases, including type 2 diabetes. Diet manipulation is the foundation of prevention and treatment of obesity and diabetes. The molecular mechanisms that mediate the diet-based prevention of insulin resistance, however, remain to be identified. Here, we report that treatment with orally administered ginger-derived nanoparticles (GDNP) prevents insulin resistance by restoring homeostasis in gut epithelial Foxa2 mediated signaling in mice fed a high-fat diet (HFD).

**Methods:** Ginger-derived nanoparticles (GDNP) were added into drinking water to treat high-fat diet fed mice for at least one year or throughout their life span. A micro array profile of intestinal, liver and fat tissue of GDNP treated mice was used to analyze their gene expression profile. Genes associated with metabolism or insulin signaling were further quantified using the real time polymerase chain reaction (RT-PCR). Surface plasmon resonance (SPR) was used for determining the interaction between Foxa2 protein and phosphatic acid lipid nanoparticles.

**Results:** HFD-feeding inhibited the expression of Foxa2; the GDNPs increased the expression of Foxa2 and protected Foxa2 against Akt-1 mediated phosphorylation and subsequent inactivation of Foxa2. Increasing expression of Foxa2 leads to altering the composition of intestinal epithelial cell (IEC) exosomes of mice fed a HFD and prevents IEC exosome mediated insulin resistance. Collectively, oral administration of GDNP prevents insulin resistance in HFD mice. Interestingly, oral administration of GDNP also extended the life span of the mice and inhibited skin inflammation.

**Conclusion:** Our findings showed that GDNP treatment can prevent HFD-induced obesity and insulin resistance via protecting the Foxa2 from Akt-1 mediated phosphorylation. GDNP treatment provides an alternative approach based on diet manipulation for the development of therapeutic interventions for obesity.

## Introduction

Obesity is a complex, multifactorial disease, affecting over a third of the world's population today. The worldwide prevalence of obesity has more than doubled in the past decade. More than 1.9 billion adults are overweight and over 600 million are obese. It is predicted that by 2030 an estimated 38% of the world's adult population will be overweight and 20% will be obese 1. Direct costs of being overweight and/or obese on medical expenses combine for approximately 5.0-10% of the United States healthcare spending 2. Therefore, obesity is considered a growing epidemic that is associated with complications, including hyperglycemia, insulin resistance, and dyslipidemia, collectively referred to as metabolic syndrome. Metabolic syndrome is a state of low-grade inflammation that supports the development of chronic diseases such as type 2 diabetes (T2D) mellitus. People with T2D develop insulin resistance and associated chronic inflammation in metabolic tissues such as adipose tissue and the liver. Recently, growing evidence has implicated intestinal tissue as an important contributor to metabolic syndrome with increased circulating levels of tumor necrosis factor-α (TNF-α), interleukin-1β (IL-1β), and IL-6 in obese humans and in diet-induced obese (DIO) mice 3, 4. Insulin resistance is a hallmark of T2D. Therefore, identifying the root cause of insulin resistance is a developing avenue for prevention of insulin resistance.

Insulin resistance occurs when cells in muscles, body fat and liver resist or ignore the response to insulin released from the pancreas, leading to high blood glucose. The role of the insulin receptor (IR) mediated pathways, including forkhead transcription factor (Foxa2) regulated pathway, have been characterized in the liver, adipose tissue, and muscular tissue 5.

In normal mice, plasma insulin inhibits Foxa2 by nuclear exclusion and in the fasted (low insulin) state Foxa2 activates transcriptional programs of lipid metabolism and ketogenesis. In insulin-resistant mice, Foxa2 is inactive and permanently located in the cytoplasm of hepatocytes. Chronic hyperinsulinemia in insulin-resistant syndrome results in the cytoplasmic localization and inactivation of Foxa2 6, 7, thereby promoting lipid accumulation and insulin resistance in the liver. Foxa2 is regulated via insulin/PI3K/AKT-mediated phosphorylation at threonine 156 (Thr156) residue 8. Phosphorylation at the Thr156 site of Foxa2 attenuates its activity and triggers nuclear export 8. Moreover, a previous study found that Foxa2 expression is an important determinant in preventing disease onset and decreasing its severity 9. Tissue-specific deletion of Foxa2 in pancreatic β cells in mice led to increased adiposity under *high*-*fat diet* conditions and decreased adipocyte glucose uptake and glycolysis 5. The role of intestinal epithelial Foxa2 in high-fat diet induced insulin resistance has not been studied.

The intestinal epithelium is a single layer of cells lining the luminal surface of the intestine that is responsible for nutrient digestion, absorption, and intestinal barrier functions. Dysregulating intestinal barrier function causes gut inflammatory mediators such as LPS 10-12 to get to the liver and other organs and initiates low grade inflammation. Collectively, these findings have moved the field forward and highlight a number of questions that need to be addressed. Specifically, in this study, using our understanding of intestine/liver crosstalk, we address whether new therapeutic strategies for prevention of high-fat diet induced insulin resistance via targeting Foxa2 are possible.

Diet manipulation is the basis of prevention and treatment of obesity and diabetes. The molecular mechanisms that mediate the diet-derived factors that prevent gut inflammation and insulin resistance remain to be identified. Ginger belongs to the family Zingiberaceae and it is one of the most widely consumed spices worldwide 13. Ginger has a long history of use as an herbal medicine to treat a variety of diseases. Most recently it was also reported that ginger has anti-inflammatory and anti-oxidative properties 14. Oral administration of ginger exosome-like nanoparticles inhibits DSS-induced mouse colitis by targeting gut microbiota 15. Whether diet-derived factors regulate the activity of Foxa2 is unknown. Given that among the numerous factors from a diet or dietary supplements that could contribute to modulate insulin signaling, identifying specific diet-derived factor(s) that contribute to modulating insulin signaling is challenging.

Edible plant-derived nanoparticles (DNPs) have only recently been reported in the therapeutic field and are known to be similar to mammalian extracellular microvesicles in terms of properties such as size distribution, surface electric charge, morphology, density, and certain components 16, 17. DNPs function as natural nanocarriers and can be used as transportation vesicles. The structural and functional biomolecules that DNPs contain can also have useful clinical applications 18, all of which have fostered the concept that these nanovesicles may be highly proficient in the development of next-generation biotherapeutic and drug delivery nanoplatforms to meet the ever-stringent demands of current clinical challenges.

In this study, we used ginger-derived nanoparticle (GDNP) as proof-of-concept to study the GDNP effect on gut epithelial Foxa2 expression in mice fed a HFD. Oral administration of GDNP to HFD-fed mice showed improved glucose tolerance and insulin response. Moreover, we found that HFD feeding inhibited the Foxa2 expression, and gut epithelial cell uptake of GDNP prevented HFD-mediated inhibition of Foxa2 expression and signaling.

## Materials and Methods

### Mice

6- to 8- week-old male C57BL/6 mice were purchased from the Jackson Laboratory (Bar Harbor, ME) and maintained on a 12-h/12-h light/dark cycle in a pathogen-free animal facility at the University of Louisville. Animal care was performed following the Institute for Laboratory Animal Research (ILAR) guidelines, and all animal experiments were conducted in accordance with protocols approved by the University of Louisville Institutional Animal Care and Use Committee (Louisville, KY).

### Cells

Murine colon (MC-38) and human colon (Caco-2) cell lines were purchased from American Type Culture Collection (ATCC) and were grown in tissue culture plate/dishes with Dulbecco Modified Eagle Medium (DMEM, Thermo Fisher Sci.) supplemented with 10% heat-inactivated fetal bovine serum (FBS), 100 U ml^-1^ penicillin, and 100 mg ml^-1^ streptomycin at 37 ºC in a 5% CO_2_ atmosphere.

### Isolation and purification of ginger-derived nanoparticles (GDNP)

Hawaiian ginger roots were purchased from a local market and the skin was peeled off manually. The skinned ginger was chopped into small pieces and mechanically blended, the juice collected and diluted in PBS. The diluted ginger juice was differentially centrifuged (500 g for 10 min, 2,000 g for 20 min, 5,000 g for 30 min, 10,000 g for 1 h) and the nanoparticles then purified on a sucrose gradient (8, 30, 45 and 60% sucrose in 20 mM Tris-Cl, pH 7.2). The band collecting at the 30% sucrose layer was carefully removed and re-purified by washing with PBS. Nanoparticle concentration and size distribution were conducted using the nanoparticle tracking analysis method provided by the Malvern NanoSight NS300 (Malvern Instruments Ltd, Malvern, United Kingdom) 19. The purified GDNPs were prepared for transmission electron microscope (TEM) using a conventional procedure and observed using a FEI Tecnai F20 at the UAB (University of Alabama, Alabama, USA) electron microscopy facility.

### Bio-distribution targeting of orally administrated GDNP by live imaging and confocal microscopy

After 6 h of orally administering 50 mg of either DiR or PKH26 fluorescent dye (Sigma) labelled GDNP, mice were sacrificed and the small intestine, colon, MLN, spleen and liver tissues were collected for imaging. DiR fluorescent signal was detected and measured using the Imaging Station Pearl Impulse (*Li-COR* Biosciences). The labeled GDNP in the gut of mice were visualized using an Odyssey CLx Imaging System (*Li-COR* Biosciences). PKH26 signal in frozen tissue sections was observed using a confocal laser scanning microscopy system (Nikon, Melville, NY). The method was previously described 14. For fat uptake, nanoparticles were isolated from the high-fat diet-fed mice using our exosome isolation protocol 20. Nanoparticles were labeled with DIR and orally administered to mice. After 6 h of oral administration, mice were sacrificed, the intestines harvested, flushed twice with PBS and scanned for DIR signals.

### High-fat diet mouse model

6 to 8-week-old C57BL/6 male mice (*n*=10 per group) were fed either a regular chow diet (RCD; 10% Fat) or a high-fat diet (HFD; 60% fat; detail description in Table 1). One HFD fed group was treated with PBS and the other HFD group was treated with GDNP (6 x 10^8^/mL) in the drinking water for at least 12 months or their entire life span. Glucose and insulin tolerance tests (GTT & ITT) were performed at 3, 6, 9 & 12 months after treatment. The data presented in this manuscript is from a minimum of 12 months of treatment.

### Lipid extraction from GDNP

Total lipids were extracted from the sucrose purified/washed band of processed ginger-derived nanoparticles 14. Briefly, 1.9 ml of a 2:1 (v/v) methanol: chloroform mixture was added to 0.5 ml of GDNPs in PBS. To this mixture 0.625 ml of each chloroform and water were added sequentially and vortexed thoroughly. The aqueous and organic phase were separated by centrifugation at 2,000 r.p.m. for 10 min at 22 ºC in glass tubes. The organic phase was collected using a glass pipette and dispensed into fresh glass tubes. The organic phase was dried under nitrogen (2 psi).

### Thin-layer chromatography (TLC) analysis

Total lipids from GDNP were quantitatively analyzed using a method previously described 14 and used for TLC analysis 18. Briefly, after extracting samples of concentrated lipid from GDNP, the lipids (PA and PC from Avanti Polar Lipids, Inc. were used as standards) were separated on a plate that had been developed with chloroform/methanol/acetic acid (190:9:1, by vol). After drying in air, the plates were stained either by iodine powder fumes or sprayed with a 10% copper sulfate and 8% phosphoric acid solution and then charred by heating at 120 °C for 12 min or until bands were developed.

### Lipidomic analysis with mass spectrometry (MS)

Extracted total lipids from GDNP or lipid band 1 (LB1) were submitted to the Lipidomics Research Center, Kansas State University (Manhattan, KS) for analysis using MS 15. In brief, the lipid composition was determined using triple quadrupole MS (Applied Biosystems Q-TRAP, Applied Biosystems, Foster City, CA). The data are reported as the concentration (nM) after normalization of the signals to internal standards of the same lipid class.

### Nanoparticle preparation from lipid extracted from GDNP

To prepare GDNP nanoparticles, GDNP-derived lipids were extracted with chloroform and dried under vacuum 15. 300 nM of lipid was suspended in 600 μl of 155 mM NaCl with or without microRNA (miR-375) or scramble RNA (20 nM each). Four μl of PEI were added followed by ultra-sonication of the mixture/solution for 20 minutes in a bath sonicator (FS60 bath sonicator, Fisher Scientific, Pittsburg, PA) at 4 ºC.

### Particle size and surface charge analysis

The particle size and zeta potential were measured using a Zetasizer Nano S90 as previously described 21.

### Affymetrix mRNA microarray

Total RNA was extracted from tissues using a Qiagen RNeasy mini kit (cat. no. 74104). One hundred ng of RNA for each sample were submitted to the Invitorgen/ThermoFisher Scientific Affymetrix facility, Santa Carla, CA, USA. The Transcriptome Analysis Console (TAC) 4.0 from ThermoFisher Scientific was used to analyze the data.

### Cytoplasmic and nuclear protein extraction

To prepare nuclear protein extracts, MC-38 cells were washed with cold PBS 14. After washing with cold PBS for 4 min, the cell pellets were re-suspended in cold cytoplasmic extract buffer (10 mM HEPES, 60 mM KCl, 1 mM EDTA, 1 mM DTT and 1 mM PMSF, pH 7.6) containing 0.075% (v/v) NP40. After incubating on ice for 3 min, the cell suspension was centrifuged at 400 g for 4 min, the supernatant (cytoplasmic protein) was collected, and the pellet was washed one more time in cytoplasmic extract buffer that did not contain NP40. Nuclear protein was extracted from the pellet with nuclear extract buffer (20 mM Tris Cl, 420 mM NaCl, 1.5 mM MgCl_2_, 0.2 mM EDTA, 1 mM PMSF and 25% (v/v) glycerol, pH 8.0). The proteins were quantified using a method as described elsewhere 14.

### Quantitative reverse transcription polymerase chain reaction (qPCR) analysis mRNA expression

Total RNA was isolated from tissue and cells using the RNeasy mini kit (Qiagen) for analysis of Foxa2 mRNA expression. One μg of total RNA was reverse transcribed using SuperScript III reverse transcriptase (Invitrogen) and quantitation was performed using primers (Eurofins) with QuantiTect SYBR Green PCR (Qiagen). GAPDH was used for normalization. The primer sequences are listed in Table S1. qPCR was run using the BioRad CFX96 qPCR System with each reaction run in triplicate.

### Western blot analysis

Homogenized tissues or cell lysates were incubated for 1 hr at 4 °C in radio-immunoprecipitation assay (RIPA) lysis buffer containing protease inhibitor. The crude lysates were centrifuged at 14,000g for 15 min. Samples were diluted in 1x SDS sample buffer after protein concentrations estimation with the BioRad Protein Assay Reagent. Proteins were separated using 10-12% or gradient SDS-PAGE and transferred to nitrocellulose membranes (Bio-Rad). Individual proteins were detected with specific antibodies and visualized by infrared fluorescent secondary antibodies (Table S2).

### Surface plasmon resonance (SPR)

SPR experiments were conducted on an OpenSPR^TM^ (Nicoya, Lifesciences, ON, CA). Experiments were performed on a LIP-1 sensor (Nicoya, Lifesciences). Tests were run at a flow rate of 20 µl/min using HBS running buffer (20 mM HEPES, 150 mM NaCl, pH 7.4). First, the LIP-1 sensor chip was cleaned with octyl-D-glucopyranoside (40 mM) and CHAPS (20 mM). Nanoparticles (1 mg/ml) were injected on the sensor chip for 10 min until stable resonance was obtained. After immobilization of nanoparticles, the surface was blocked with BSA (3%) in running buffer. After a stable signal was obtained, recombinant human Foxa2 protein (100 nM to 1 µM; cat. no. ab95848; Abcam, USA) or synthesized peptides (500 nM to 1 µM; GenScript Biotech, USA) were run over the immobilized liposomes. A negative control was also performed by injecting protein onto a blank sensor chip to check for non-specific binding. After 10 min, the nanoparticles binding to protein were eluted using NaOH (200 µM). The sensograms were analyzed using TraceDrawer kinetic analysis software 22.

### Histological analysis

For hematoxylin and eosin (H&E) staining, tissues were fixed with buffered 10% formalin solution (SF93-20; Fisher Scientific, Fair Lawn, NJ) overnight at 4 °C. Dehydration was achieved by sequential immersion in a graded ethanol series of 70%, 80%, 95%, and 100% ethanol for 40 min each. Tissues were embedded in paraffin and subsequently cut into ultrathin slices (5 μm) using a microtome. Tissue sections were deparaffinized in xylene (Fisher), rehydrated in decreasing concentrations of ethanol in PBS, stained with H&E, and the slides were scanned with an Aperio ScanScope using a method previously described 23.

### *In vivo* intestinal permeability assay

For *in vivo* intestinal permeability studies, fluorescein-5-isothiocyanate (FITC)-conjugated dextran (MW 4000; Sigma-Aldrich, St. Louis, MO) was administered by oral gavage at a concentration of 60 mg/100 g of body weight. Blood was collected retro-orbitally two hours later, and serum was harvested. Fluorescence intensity was determined with a fluorescence spectrophotometer (BioTek) at emission and excitation wavelengths of 485 nm and 528 nm, respectively. FITC concentration was measured from standard curves generated by serial dilution of FITC-dextran 15.

### Enzyme-linked immunosorbent assay (ELISA)

Tumor necrosis factor (TNF)-α, interleukin (IL)-1β, IL-6 and IL-10 concentrations in plasma were quantified using an ELISA described previously 24. ELISA reagents were purchased from eBioscience, and assays were performed in accordance with the manufacturer's instructions. Briefly, microtiter plate wells were coated with anti-mouse TNF-α, IL-1β, IL-6 or IL-10 antibody at 1:200 overnight at 4 °C. Excess binding sites were blocked with 100 µl/well of blocking solution (PBS containing 0.5% BSA) at room temperature for 1 h. After washing three times with PBS containing 0.05% Tween 20, sera collected from mice were diluted 2-fold, added in a final volume of 50 µl to the plate wells and incubated for 1 h at 37 °C. After 3 washes with PBS, the plate was incubated with 100 µl of HRP-conjugated anti-mouse antibody (Pierce) diluted 1:50,000 in blocking solution for 1 h at RT. After the final 3 washes with PBS, the reaction was developed for 15 min, blocked with H_2_SO_4_ and optical densities were recorded at 450 nm using a microtiter plate reader (BioTek Synergy HT).

### Lipid analysis in plasma

Peripheral blood sample of mice were collected into non-heparinized capillary tubes coated with 4% sodium citrate. The levels of cholesterol and triglycerides were determined using a Piccolo lipid panel plus (Abaxis Inc, USA).

### Glucose and Insulin tolerance tests (GTT & ITT)

For glucose tolerance tests, after an overnight fast, baseline glucose levels were determined using a glucometer (Priology, USA). Mice were then given an intraperitoneal injection of glucose (dextrose) at a dose of 2 mg/g of body weight 24. The blood glucose levels were measured at 30, 60, 90, and 120 min after glucose injection. For insulin tolerance tests, mice were fasted for 6 h and basal blood glucose levels were determined. Then, mice were given an intraperitoneal injection of insulin (1.2 units/g of body weight). The blood glucose levels were measured at 30, 60, and 90 min (otherwise indicated in figures) after insulin injection.

### Confocal microscopy

For frozen sections, periodate-lysine-paraformaldehyde (PLP) fixed tissues were dehydrated with 30% sucrose in PBS overnight at 4 °C and embedded into optimal cutting temperature (OCT) compound. Tissue was subsequently cut into ultrathin slices (5 μm) using a microtome and placed on glass slides. Tissue sections were blocked with 5% bovine serum albumin (BSA) in PBS. Primary antibodies (1:800) were added onto the slides and incubated at 4 °C overnight. Sections were washed three times followed by secondary antibodies conjugated to a fluorescent dye (at 1:2000 dilution). Nuclei were stained with 4', 6-diamidino- 2-phenylindole dihydrochloride (DAPI). For in-vitro cultured cells, 2 × 10^5^ cells were grown on coverslips in six well plates and co-cultured with PKH26 labeled fecal exosomes for 16 h at 37 °C in a 5% CO_2_ incubator. Cells were washed with PBS and fixed with 2% PFA. Nuclei were stained with DAPI. Tissues and cells were visualized using confocal laser scanning microscopy 25 (Nikon, Melville, NY).

### Cytokine production in plasma & skin tissues

To investigate the effects of GDNP on the regulation of cytokine production in peripheral blood and skin tissues, peripheral blood and skin tissues from the surrounding belly area were collected from HFD fed mice treated for 12 months with PBS or GDNP. Plasma was collected from blood and tissue fluids from skin tissues. Cytokines were analyzed with a Proteome Profiler Mouse XL Cytokine Array Kit (R&D Systems, ARY028) as per the manufacturer's instructions. Quantification of the spot intensity in the arrays was conducted with background subtraction using HLImage++ (Western Vision Software).

### Statistical analysis

GraphPad Prism 7.0 (GraphPad software) was used for data analysis. The data are presented as a value ± standard deviation (mean ± SD). Student's *t*-test (two-tailed) and one-way (Bonferroni multiple comparison) or two-way ANOVA were used as appropriate. Differences were considered significant when the *P*-value was less than 0.05. *P* values >0.05 were considered not significant (NS). * < 0.05, ** < 0.01, *** < 0.001, ****<0.0001.

## Results

### Ginger-derived nanoparticles (GDNP) prevent high-fat diet-mediated inhibition of Foxa2 expression in intestinal epithelial cells (IECs)

Ginger extract administration can prevent HFD-induced obesity 26 and fructose overconsumption-induced insulin resistance in animal models 27. Given the critical role of Foxa2 in regulation of insulin signaling 6, 28-32, this observation motivated us to dissect the role and mechanism of action of this transcription factor in a high-fat diet induced obesity mouse model. First, we isolated GDNP through differential centrifugation process. Sucrose gradient-purified (Figure S1A) ginger nanoparticles from the centrifuged pellet (10,000g) were characterized using electron microscopy (Figure S1B) and a Zeta sizer. The GDNP had a mean size of 250 ± 72 nm (Figure S1C) with a yield of 1x10^12^ GDNP/g ginger tissue. The GDNP had a charge of -220 ± 131 mV as determined by the Zeta sizer (Figure S1D). The GDNP had no detectable levels of bacterial LPS (Figure S1E). Thin-layer chromatography (TLC) revealed that some lipids in GDNP are enriched (Figure S1F; red box) while others are absent (indicated by blue arrow) when compared to the lipids extracted from whole ginger root (Figure S1F). Quadrupole mass spectrometry (MS) analysis of the GDNP lipid profile (Figure S1G; Table S3) revealed that phosphatidic acid (PA) represented more than 38.0% of the lipid content, followed by 32.7% digalactosyldiacylglycerol (DGDG) and 21.3% monogalactosyldiacylglycerol (MGDG). Other lipids present in minor concentrations were PI, PC, PG and LysoPG. Further MS analysis of the TLC extracted lipid band (LB1) revealed that >74% of PA present was in LB1 followed by LysoPG, DGDG, PI and MGDG (Figure S1H; Table S3).

**Figure 1:**
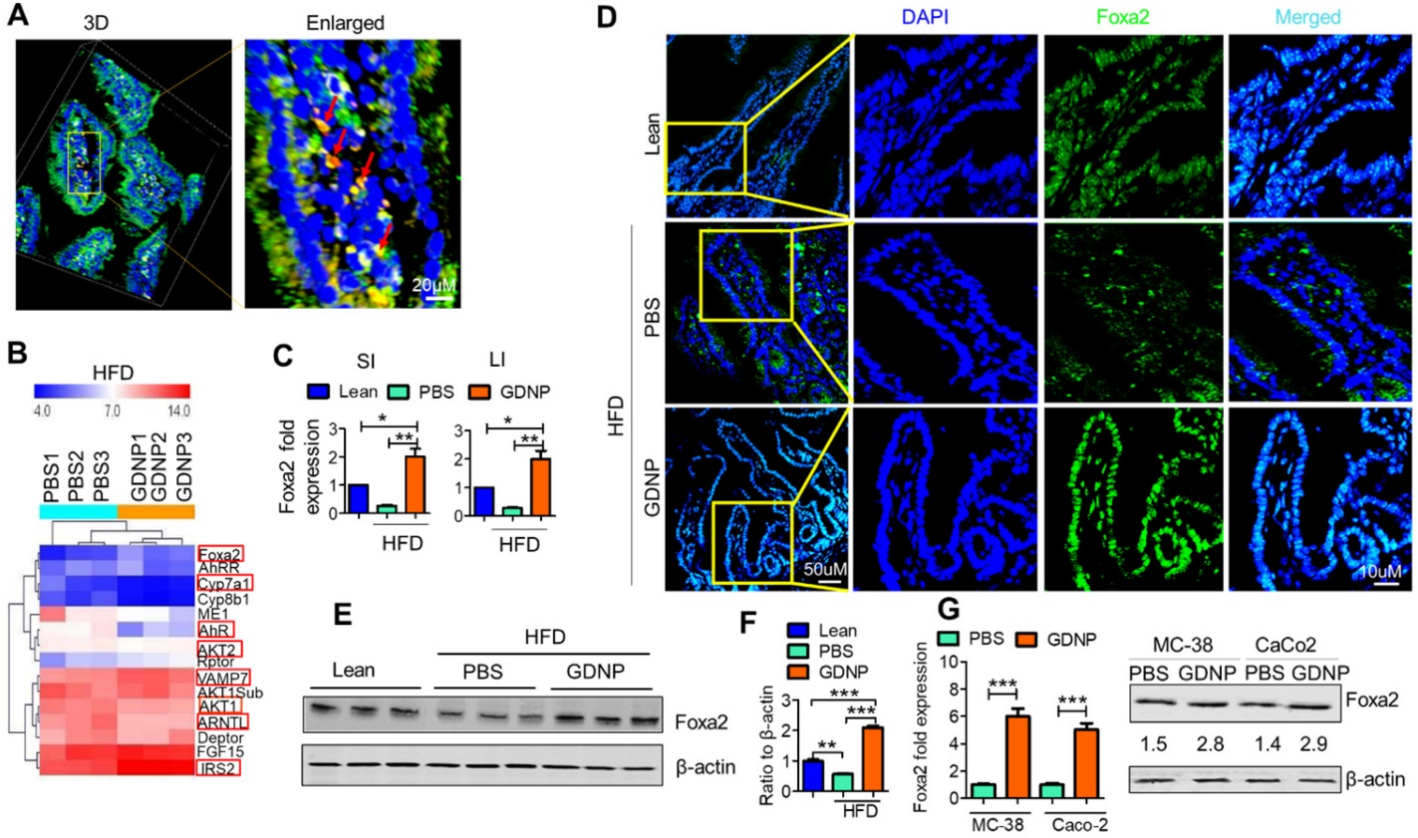
** Ginger-derived nanoparticles (GDNP) inhibit the phosphorylation of Foxa2 in intestinal epithelial cells. A.** PKH-26 (red)-labeled GDNP uptake by small intestine epithelial (A33 positive/green) cells as shown by confocal 3D imaging. Enlarged image of cells containing labeled GDNP (PKH26/red) shown by red arrows (n = 5/group). **B.** Representing the alteration of gene expression in the Affymetrix array of small intestinal (SI) tissues from high-fat diet (HFD)-fed mice treated with either PBS or GDNP. Red boxes highlight the genes involved in insulin signaling and lipid metabolism (n = 3/group). **C.** Normalized (to β-actin) qRT-PCR quantification of Foxa2 mRNA expression in the mouse small intestine (SI) and large intestine (LI) (n = 5/group). **D.** Confocal images of frozen sections of the small intestine showing Foxa2 expression (green) and DAPI for nucleus staining (blue) (n = 5/group). **E.** Western blot representing total Foxa2 expression in mouse small intestine tissues (n = 3/group). **F.** Corresponding densitometry analysis of the western blot for Foxa2 protein expression (expressed as the ratio to β-actin expression). **G.** Upregulation of Foxa2 mRNA (bar graphs, left panel) and protein (western blot, right panel) expression in GDNP-treated mouse colon (MC-38) and human colon (Caco2) cell lines. The ratio to β-actin shown in the middle (numbers). Data represent three independent experiments. One-way ANOVA with the Bonferroni correction for multiple comparisons and student *t* test (one tailed) were used to calculate statistical significance (*p* value *<0.05; **<0.01; ***<0.001).

Next, we determined the biological effects of orally administered GDNP. To do so, we added the GDNP or PBS in an equal volume as a control into drinking water. After 2 weeks of treatment, we evaluated the plasma for any cytotoxic effect of GDNP. Results from the plasma evaluation for cholesterol, HDL, triglycerides, ALT, AST, glucose, LDL and VLDL and indicated there was no significant difference between PBS and GDNP treated RCD mice (Figure S2A). Further, we labelled the GDNP with two different fluorescent dyes (DIR for live imaging and PKH-26 for confocal based analysis) and gavaged each of the mice. Live imaging of the mice suggested that GDNP were still present in the intestine at 6 h (Figure S2B). Confocal image analysis of the intestinal tissues suggested that GDNP is taken up by gut epithelial (PKH26^+^A33^+^) cells (Figure 1A).

We next investigated the effects of GDNP uptake on Foxa2 expression. Affymetrix array analysis of intestinal tissue, followed by confirmation with qPCR, revealed that GDNP treatment induced the expression of Foxa2 and modulate the expression of several other genes involved in glucose metabolism and insulin signaling (highlighted in red rectangle boxes, Figure 1B; Table S4).

The qPCR results suggested that a 12-month HFD feeding led to a decrease in the expression of Foxa2, and GDNP treatment of 12-month HFD-fed mice caused a two-fold increase in Foxa2 mRNA levels in the small and large intestinal tissues relative to lean mice (Figure 1C). Consistent with the qPCR results, confocal images (Figure 1D) and western blot analysis of small intestinal tissues (Figure 1E-F) also suggested an increase in total Foxa2 protein in HFD-fed mice treated with GDNP. This *in vivo* result was also demonstrated in an *in vitro* culture system. When mouse (MC-38) and human (Caco2) colon cells were cultured with GDNP (12 h), the Foxa2 mRNA and protein levels were upregulated (Figure 1G). Although the GDNP contains proteins and RNAs as well, the RNAs and proteins have no impact on the expression of Foxa2 (Figure S2C), So, in this study, only GDNP lipids were further studied relative to a Foxa2 mediated insulin response. Taken together, these results suggest that orally administered GDNP can prevent HFD-induced decreases in Foxa2 expression in the intestine.

### GDNP phosphatidic acid (PA) induces Foxa2 expression in intestinal epithelial cells

**Figure 2:**
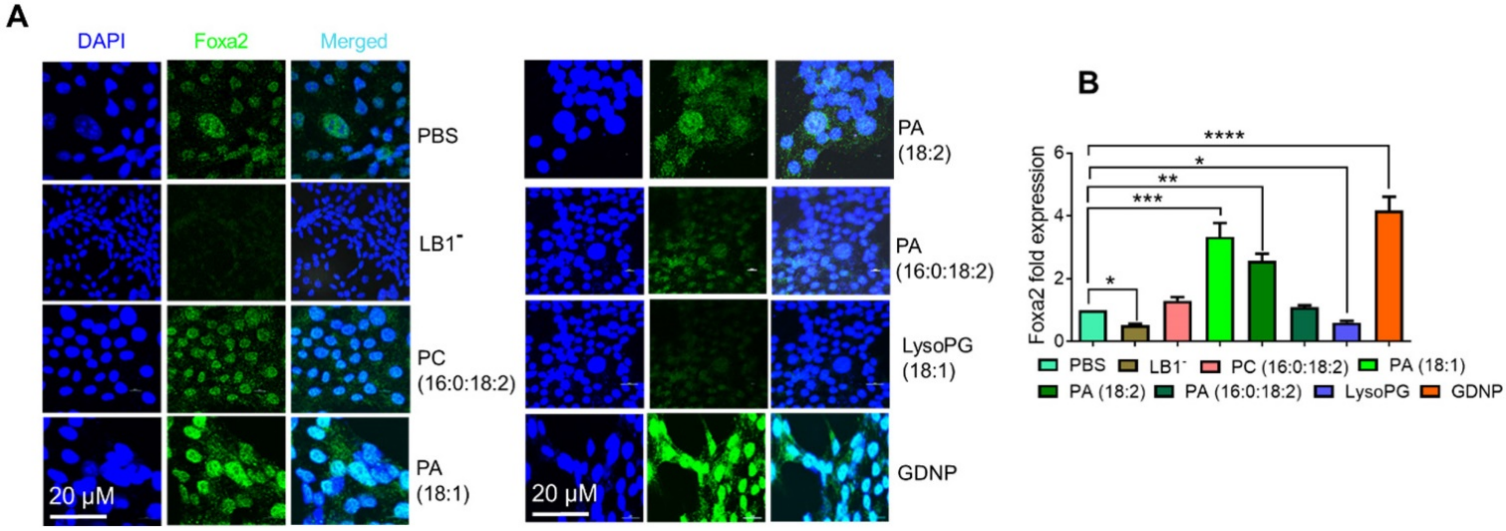
** GDNP enhances Foxa2 expression in intestinal epithelial cells. A.** Visualization of Foxa2 (green) expression in MC-38 cells cultured with different lipid nanoparticles or complete GDNP. DAPI was used for nuclear staining. LB1^-^, lipid band 1 depleted; PC, phosphatidylcholine; PA, phosphatidic acid; LysoPG, lysophosphatidylglycerol; GDNP, ginger-derived nanoparticles. **B.** qPCR quantification of Foxa2 mRNA in MC-38 cells cultured with different lipid nanoparticles or complete GDNP, as in panel a. Data represented from three independent experiments. One-way ANOVA with the Bonferroni correction for multiple comparisons was used to calculate statistical significance (*p* value *<0.05; **<0.01; ***<0.001; ****< 0.0001).

Since PA was the most enriched component of GDNP (see Figure S1H), we next hypothesized that PA might be responsible for the observed GDNP-induced upregulation of Foxa2 expression in gut epithelial cells. To test this hypothesis, we extracted the total lipids from GDNP and separated them by TLC (see Figure S1F), and each lipid band was excised and reconstituted as lipid nanoparticles. Lipid nanoparticles from LB1 extracted from the TLC plate total GDNP lipid increased Foxa2 expression (~4-fold) in MC-38 cells compared to PBS-treated cells (Figure S2D). Lipid nanoparticles made from total GDNP lipid with LB1 depleted using a technique previously described 14 abolished the induction of Foxa2 expression in these cells, suggesting that LB1 was responsible for the upregulation of Foxa2 (Figure 2). However linear lipid from ginger was not as effective as nanoparticles in terms of Foxa2 induction (Figure S2E).

To determine the specific role of PA in the upregulation of Foxa2 expression in intestinal epithelial cells, we also generated nanoparticles from commercially available PA lipids. Nanoparticles generated from LysoPG (18:1) and PC (16:0:18:2) were used as controls. Treating MC-38 cells with these lipid nanoparticles indicated that PA 18:1 and 18:2 significantly induced Foxa2 expression, whereas PA (16:0:18:2) did not affect Foxa2 expression (Figure 2A). Moreover, LysoPG nanoparticles downregulated both Foxa2 protein and mRNA expression (Figure 2B). Collectively, these results confirmed that the GDNP PA was largely responsible for the GDNP-induced upregulation of Foxa2 expression in intestinal epithelial cells.

### GDNP PA prevents phosphorylation of Foxa2 by inhibiting Akt-1 activation

**Figure 3:**
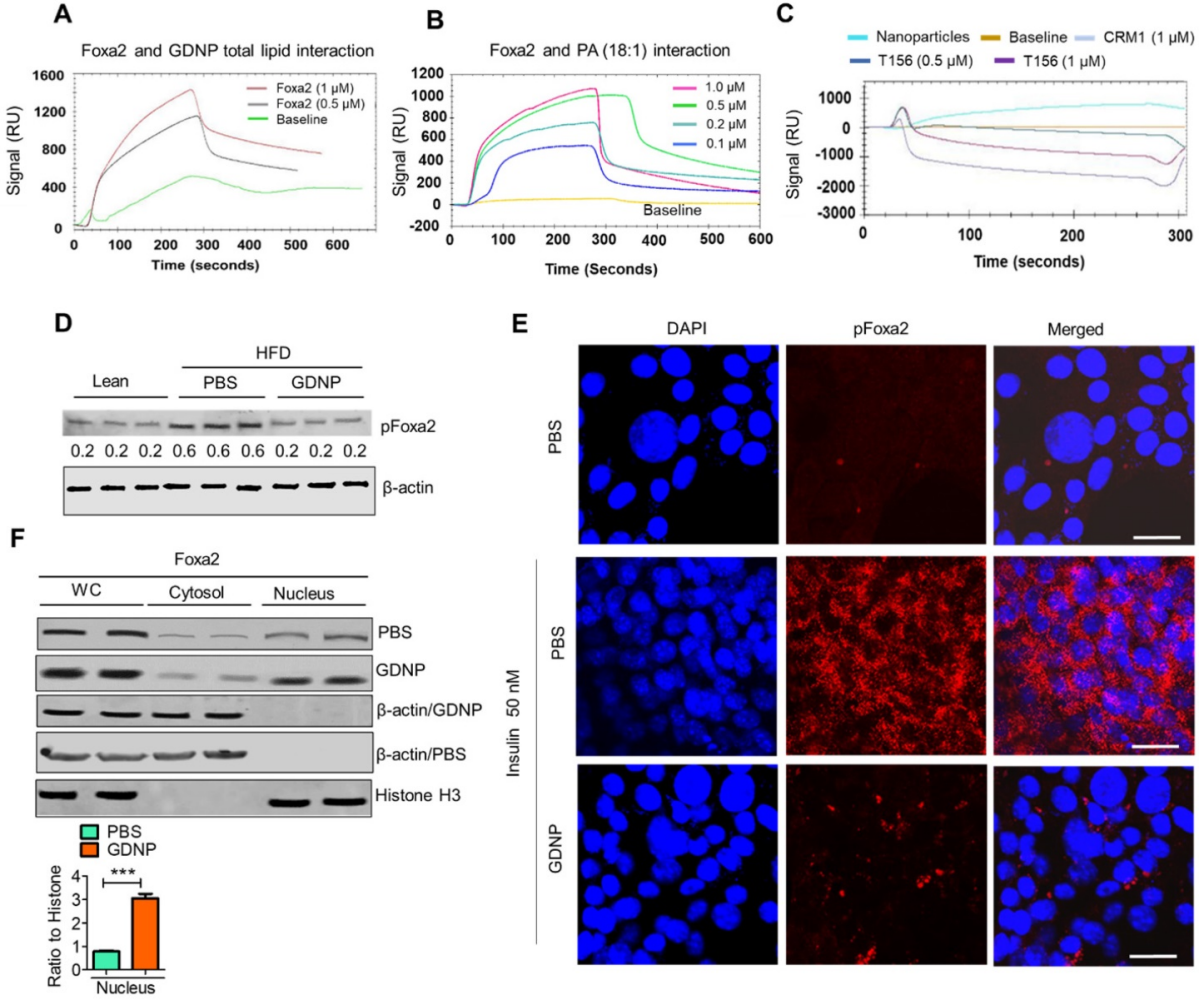
** Phosphatic acid protects Foxa2 from Akt-1 mediated phosphorylation by covering Thr156. A & B.** The surface plasmon resonance (SPR) sensogram (response unit) representing the interaction between GDNP lipid nanoparticles (**A**) and phosphatidic acid (PA 18:1) nanoparticles (**B**) with recombinant Foxa2 protein. **C.** The SPR sensogram represents the interaction between PA (18:1) nanoparticles and the CRM1 and Thr156 Foxa2 synthesized peptide sequences. **D.** Western blot of phosphorylated Foxa2 (pFoxa2) expression in small intestinal tissue derived from lean and HFD mice (n = 3/group). The ratio to β-actin shown in the middle (numbers). **E.** Confocal images showing expression of pFoxa2 (red) in MC-38 cells cultured with 50 nM insulin and PBS or GDNP. Scale bar is 20 μM. **F.** Western blot of whole cell lysate (WC), nuclear and cytoplasmic levels of Foxa2 in MC-38 cells treated with PBS or GDNP. The ratio to histone for nuclear expression of Foxa2 shown on the right. Student *t* test (one tailed) were used to calculate statistical significance (*p* value ***<0.001).

We next hypothesized that the PA of GDNP not only induces the expression of Foxa2 in intestinal cells but also enhances the activity of Foxa2. Therefore we began exploring the potential interaction of GDNP lipids, in particular PA, with Foxa2 using surface plasmon resonance (SPR) which is an optical technique utilized for detecting molecular interactions 33. We coated GDNP total lipids on the LIP-1 sensor chip containing a covalently attached lipophilic group to determine whether GDNP lipids interact with recombinant Foxa2 protein. We identified a SPR sensogram peak indicating that GDNP total lipids showed a strong interaction with recombinant Foxa2 protein (Figure 3A). Lipid nanoparticles made from commercially available PA (18:1) were coated on the LIP-1 sensor and recombinant Foxa2 was run over the sensor. Consistent with the GDNP total lipid results, Foxa2 recombinant protein also showed a strong interaction with PA (18:1) nanoparticles, and the strength of this interaction was found to be Foxa2 protein concentration-dependent (Figure 3B). We then determined the PA-binding site of Foxa2. It has been suggested that phosphorylation of Foxa2 at Thr156 results in its translocation from the nucleus to the cytoplasm, which in turn leads to its inactivation 6. Another signal sequence for nuclear exclusion, called CRM1, has also been reported 34. Thr156 and CRM1 protein peptides were designed and run over the PA nanoparticle coated LIP-1 sensor. SPR sensograms from the testing of these peptides suggest that peptide Thr156 interacts with PA, whereas the CRM1 peptide did not show any notable interactions (Figure 3C). Altogether, these results confirmed that PA from GDNP binds to Foxa2, potentially at Thr156.

Phosphorylated Foxa2 (pFoxa2) is translocated from the nucleus to the cytoplasm during insulin exposure (hyperinsulinemia) and results in the inactivation of Foxa2, and it remains permanently inactive in conditions characterized by insulin resistance, such as T2D 6. Thus, we next determined whether GDNP regulates the insulin-mediated phosphorylation of Foxa2. Relative to lean mice, PBS-treated HFD-fed mice had significantly elevated levels of pFoxa2 in the small intestinal tissue. Treatment of HFD-fed mice with GDNP, however, led to a reduction in small intestine pFoxa2 levels relative to the PBS-treated animals (Figure 3D), suggesting that GDNP treatment inhibits the phosphorylation of Foxa2. This *in vivo* result was further confirmed in MC-38 cells, since cells grown in the presence of insulin (50 nM) showed high levels of cytoplasmic pFoxa2 relative to control cells, but co-treatment with insulin and GDNP resulted in reduced levels of pFoxa2 relative to treatment with insulin alone (Figure 3E).

Previous studies have shown that phosphorylation of Foxa2 is mediated by pAkt-1 6, which itself is phosphorylated by mTOR. We thus examined whether GDNP treatment inhibited the expression of pAkt-1 and mTOR. Indeed, pAkt-1 expression was also reduced in MC-38 cells grown in the presence of insulin (50 nM) relative to controls; co-culture with insulin and GDNP (12 h) reduced the expression of pAkt-1 (Figure S3A). Moreover, the levels of mTOR and pFoxa2, but not the pAkt-2 levels, were decreased in GDNP-treated MC-38 cells compared to PBS-treated MC-38 cells grown in the presence of insulin for 12 h (Figure S3B). Finally, GDNP-treated MC-38 cells showed increased expression of Foxa2 in the nucleus compared to PBS-treated MC-38 cells (Figure 3F). Collectively, these results suggest that GDNP treatment blocks insulin-mediated phosphorylation of Foxa2 via the inhibition of pAkt-1.

### GDNP mediated altering of the lipid composition of intestinal epithelial exosomes contributes to preventing high-fat diet induced glucose intolerance and insulin resistance

**Figure 4:**
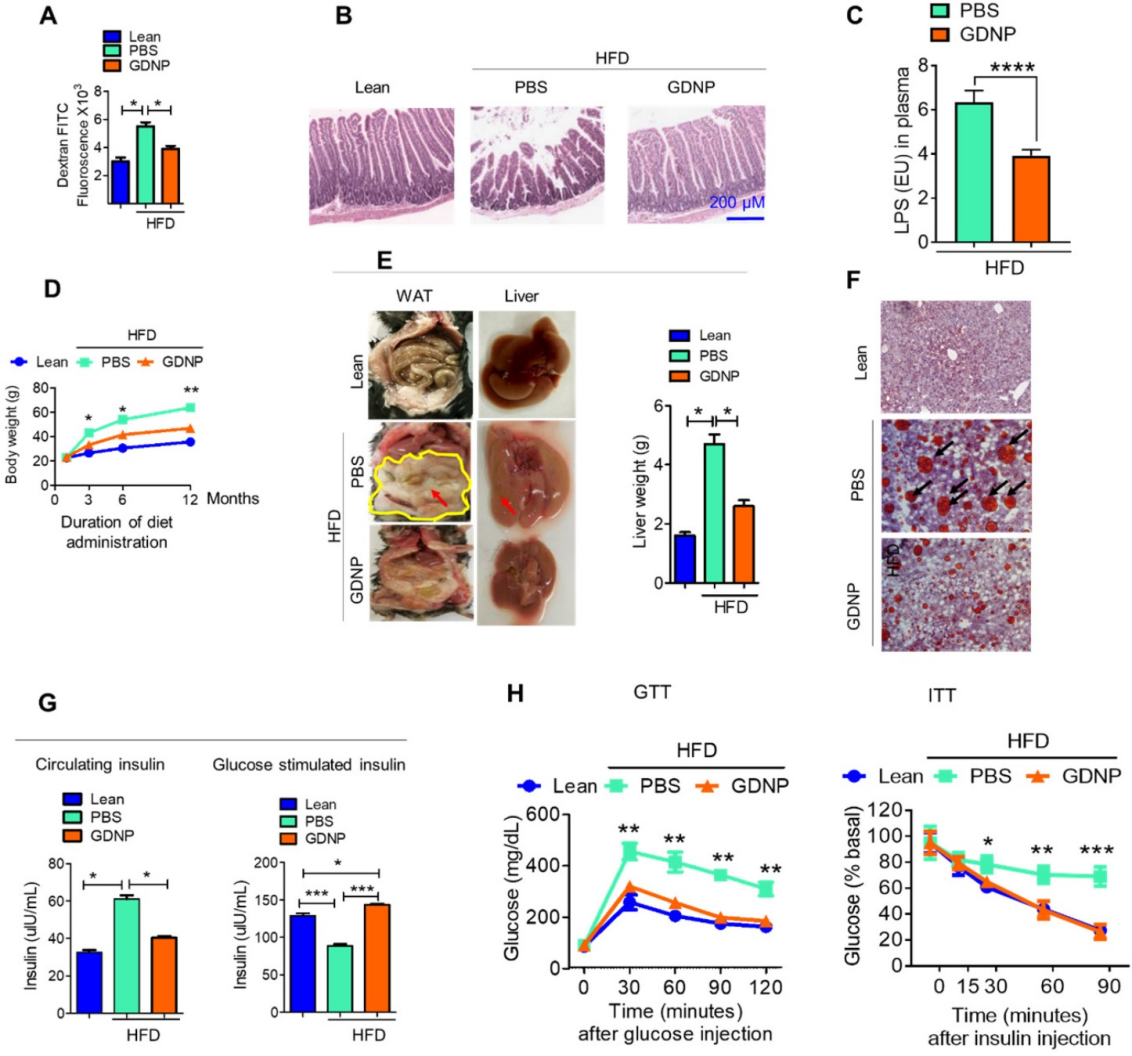
** GDNP prevents the development of HFD-induced glucose intolerance, insulin resistance, inflammation, and decrease in life span. A.** Quantification of plasma dextran FITC fluorescence in lean and HFD-fed mice treated with PBS and GDNP to determine the gut permeability (n = 5/group). **B.** H & E staining of small intestinal tissues from lean, and PBS- and GDNP-treated HFD-fed mice (n = 5/group). **C.** Levels of circulating LPS in plasma (n=5/group). **D.** Body weights at various time points of diet administration (RCD or HFD). Statistical significance was calculated between PBS- and GDNP-treated HFD-fed mice (n = 5/group). **E.** Images of the white adipose tissue (WAT) and liver in lean and PBS- or GDNP-treated HFD-fed mice. Fat deposition shown by red arrows. Liver weight after 12 months of PBS or GDNP treatment (n = 5/group). **F.** Oil red O staining of liver tissue derived from 12 months of PBS or GDNP treatment (n = 5/group). **G.** Quantification of levels of circulating insulin (left panel) and glucose-induced insulin (right panel) in lean and PBS- or GDNP-treated HFD-fed mice (n = 5/group). **H.** Glucose tolerance test (GTT) and insulin tolerance test (ITT) of lean and HFD-fed mice treated with PBS or GDNP at 12 months. One-way ANOVA with Bonferroni post hoc test was used for statistical significance (n = 5/group). One-way ANOVA with the Bonferroni correction for multiple comparisons or Student t test was used to calculate statistical significance (*p* value *<0.05; **<0.01; ***<0.001).

The liver is a metabolic organ and plays a critical role in regulating glucose tolerance and insulin responses. Gut permeability is a critical factor for regulating glucose tolerance and insulin responses via the gut/liver axis 35. A HFD is known to increase gut permeability 36 which in turn contributes to the development of insulin resistance. Because of this, the link between GDNP mediating an increased expression of intestinal epithelial cell Foxa2 and insulin resistance was further investigated. First, we determined whether gut permeability was altered in HFD-fed mice treated with GDNP (>12 months) using dextran FITC. The dextran FITC results suggested that HFD-fed mice receiving PBS had elevated plasma levels of dextran FITC compared to GDNP-treated HFD-fed mice and lean mice, suggesting that the elevated levels of dextran FITC in the plasma was due to increased gut permeability (Figure 4A). Further we evaluated the expression of tight junction proteins such as occludin, claudin and ZO-1 in small intestinal tissues of high-fat diet fed mice treated with PBS or GDNP. Results suggested that GDNP treatment inhibits the HFD mediated downregulation of the above-mentioned tight junction proteins (Figure S4A - C). Moreover, histological (H & E staining) analysis of the small intestine indicated that HFD-fed mice that received GDNP for >12 months showed normal intestinal integrity, similar to that of lean mice. Whereas HFD-fed mice that received PBS showed disrupted villi, which is indicative of compromised gut integrity (Figure 4B). Increasing levels of LPS circulating in blood due to increasing gut permeability plays a role in inducing systemic chronic inflammation 37, 10-12. We further quantified the circulating levels of LPS in plasma derived from HFD-fed mice treated with PBS or GDNP. Results suggest that mice receiving the GDNP in drinking water have significantly low levels of circulating LPS compared to PBS treated mice (Figure 4C).

We next tested whether oral administration of GDNP prevents HFD-induced glucose intolerance and insulin resistance, by administering GDNP in drinking water for 12 months. Our data suggests that GDNP treatment of HFD-fed mice prevented the HFD-induced increases in body weight, liver weight, and white adipose tissue (Figure 4D-F). GDNP also prevented the development of hyperinsulinemia (Figure 4G). In the HFD-fed mice the secretion of insulin from pancreatic tissue was more in the GDNP treated group compared to the PBS group (Figure S4D). However, there was no significant difference in glucagon expression between these two groups (Figure S4E). We also noticed that GDNP treated mice exhibited less food intake as well (Figure S4F). This result was further supported by less fat uptake as HFD-derived nanoparticles (Figure S4G) and had lower insulin resistance indices (Figure S4H).

**Figure 5:**
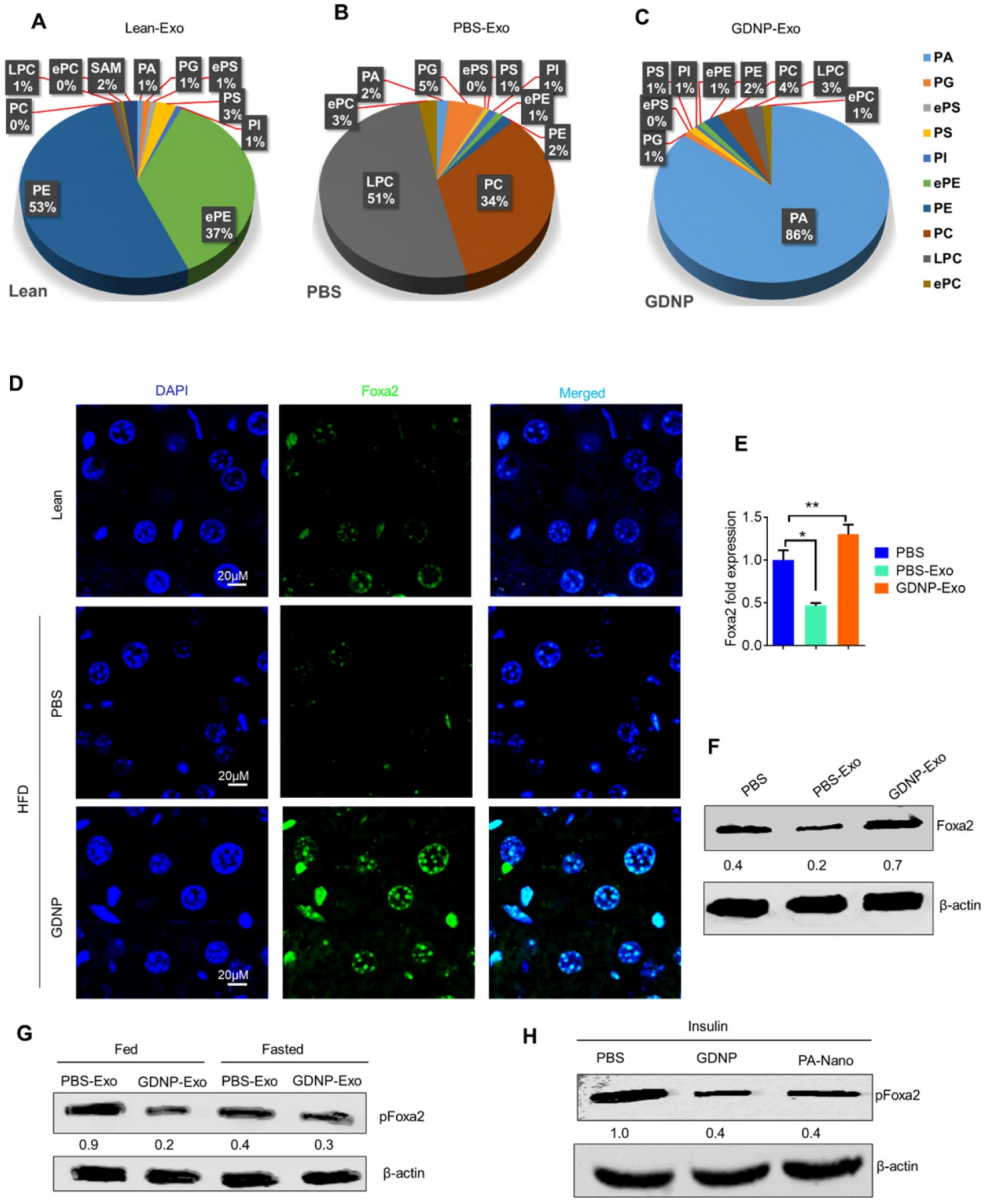
** GDNP treatment enhances the Foxa2 expression in liver. A.** Pie graphs represents targeted lipidomics analysis of phospholipids in IEC exosomes derived from lean mice after 12 months at RCD (n =5/group). **B.** Pie graphs represents targeted lipidomics analysis of phospholipids in IEC exosomes derived from HFD-fed mice receiving PBS for 12 months (n =5/group). **C.** Pie graphs represents targeted lipidomics analysis of phospholipids in IEC exosomes derived from HFD-fed mice receiving GDNP for 12 months (n =5/group). **D.** Confocal images represent Foxa2 expression in liver sections from lean mice and HFD-fed mice receiving PBS or GDNP for 12 months (n =5/group). **E.** qPCR for Foxa2 mRNA expression in primary hepatocytes cultured with exosomes for 16 hours. Data represented from three independent experiments. **F.** Western blots for Foxa2 protein in primary hepatocytes cultured with exosomes for 16 hours. Ratio to β-actin shown in the middle (numbers). Data represented from three independent experiments. **G.** Western blots for pFoxa2 protein in primary hepatocytes cultured with exosomes derived from HFD-fed mice for 16 hours in fed and fasted condition. The ratio to β-actin shown in the middle (numbers). **H.** Western blots for pFoxa2 protein in primary hepatocytes cultured with GDNP and nanoparticles made up from phosphatic acid (PA-Nano) for 16 hours in the presence of insulin (50 nm). The ratio to β-actin shown in the middle (numbers). One-way ANOVA with the Bonferroni correction for multiple comparisons was used to calculate statistical significance (*p* value *<0.05; **<0.01).

GDNP treated HFD-fed mice exhibited a protection against the development of glucose intolerance and insulin resistance (Figure 4H). Crosstalk between gut/liver regulates insulin signaling in the liver. Next, we determined the mediator that regulates cross-talk between the liver and gut in high-fat diet induced insulin resistance. Exosomes are known to play a critical role in intercellular communications 38. Our published data indicate that exosomes released from intestinal epithelial cells (IEC) of HFD-fed mice can traffic to the liver and inactivate the insulin pathway in hepatocytes 20. Lipids of IEC exosomes from HFD-fed mice cause mice to develop insulin resistance in a gut microbiota independent manner 20. Therefore, instead of further investigating whether IEC exosomes from GDNP treated HFD-fed mice play a different role in modulating the liver insulin signaling pathway via gut microbiota as we had done in a previous study 20, we explored whether oral administration of GDNP in drinking water can alter the composition of IEC exosomal (CD63^+^A33^+^) lipids. Quadrupole MS analysis (targeted lipidomic analysis of phospholipids) of lipid content of exosomes released from IEC of mice on an RCD have a lipid content of ~90% phosphatidylethanolamine (PE; Figure 5A). However, long-term feeding of the HFD led to an increase in the phosphatidylcholine (PC) as shown in Figure 5B. GDNP treatment led to an increase of phosphatic acid (~86%) compared to PBS treatment in HFD-fed mice. Moreover, there was significant reduction in phosphatidylcholine (PC) in exosomes derived from GDNP treated HFD-fed mice compared to PBS treated mice (Figure 5C). GDNP treatment provided protection against the HFD mediated downregulation/inactivation of Foxa2 in the liver (Figure 5D) and adipose tissue (Figure S5A). However, no significant difference was seen in the expression of the insulin receptor in adipose tissue (Figure S5B). Published data 20 suggest that intestinal epithelial cell IEC-derived exosomes traffic to the liver and are taken up by hepatocytes, and the reduction of exosomal PE and increase of exosomal PC contributes to development of the insulin resistance 20. To prove that IEC exosomes have an impact on Foxa2 expression in hepatocytes, we cultured primary hepatocytes for 16 h with intestinal epithelial released (CD63^+^A33^+^) exosomes isolated from HFD-fed mice treated with GDNP (GDNP-Exo) or PBS (PBS-Exo) as a control in drinking water for 12 months. Expression of Foxa2 mRNA by qPCR and protein products were quantified by western blots. These results revealed that GDNP-Exo treatment increased the Foxa2 mRNA and protein levels as well (Figure 5E-F).

Western blots analysis further indicates that GDNP-Exo prevented the Foxa2 from phosphorylation, however, PBS-Exo were not able to prevent the Foxa2 phosphorylation (Figure 5G) in the HFD-fed mice. Further, to determine whether PA can protect Foxa2 from being phosphorylated, primary hepatocytes were treated with nanoparticles generated from synthesized PA (Avanti Inc.) or GDNP and the level of pFoxa2 was quantified by western blots. The results suggested that PA nanoparticles were capable of preventing Foxa2 phosphorylation (Figure 5H).

### GDNP administration protects HFD-fed mice from chronic skin inflammation and improves the life span of mice

**Figure 6:**
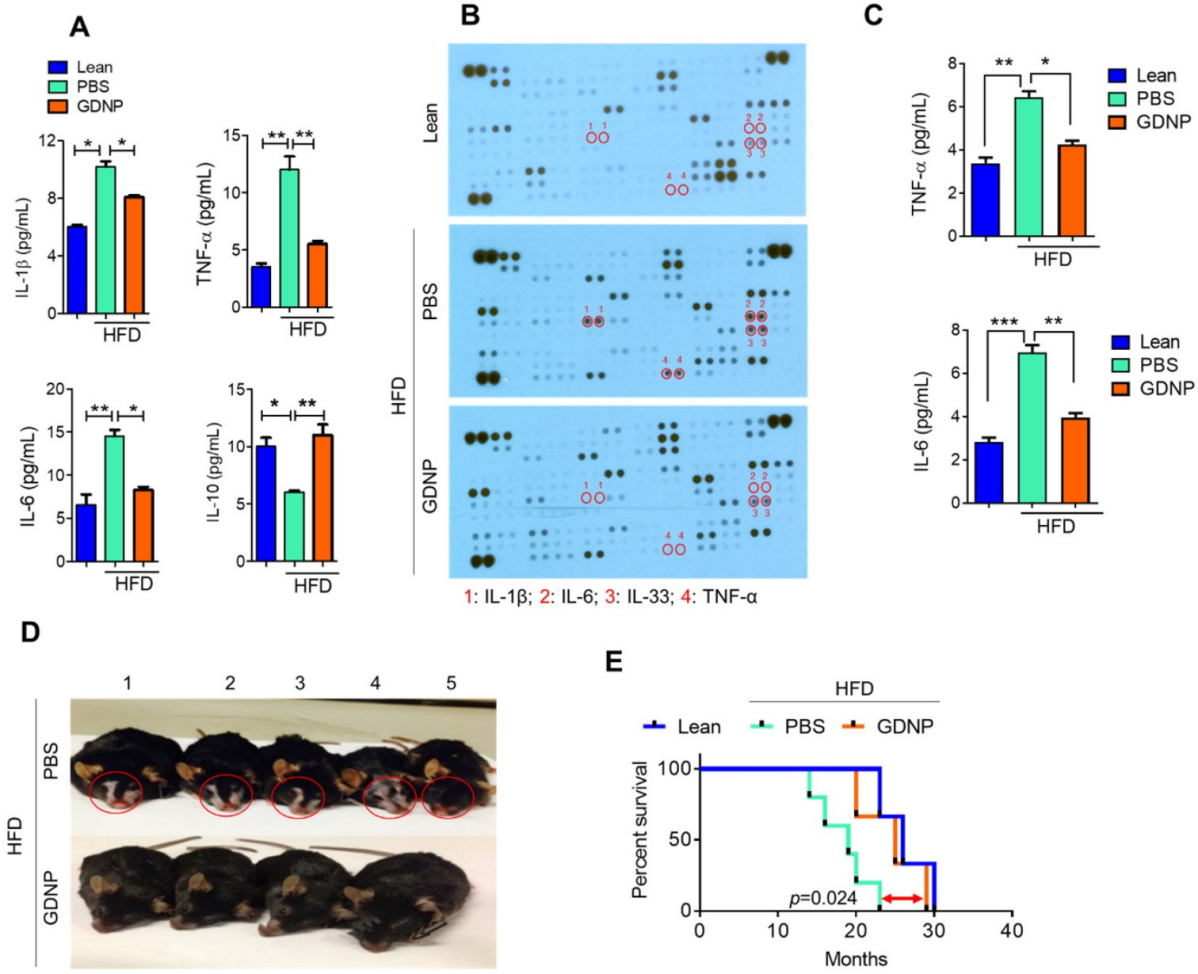
** GDNP inhibited HFD mediated chronic inflammation and improves the life span of mice. A.** Quantification of plasma levels of pro-inflammatory (IL-1β, IL-6 and TNF-α) and anti-inflammatory (IL-10) cytokines in lean and PBS- or GDNP-treated HFD-fed mice (n = 5/group). **B.** Cytokine array for skin tissue obtained from lean and PBS- or GDNP-treated HFD-fed mice. Pro-inflammatory cytokines spots/positions are indicated with red circles (n = 5/group). **C.** Quantification of inflammatory cytokines TNF-α and IL-6 by ELISA in skin tissues (n = 5/group). **D.** Representative images of the phenotypic changes induced by 12 months of HFD feeding. Note the changes in skin/fur color (red circle) and hair loss in PBS-treated HFD mice (upper row) compared to GDNP treated mice (lower row). **E.** Percentage survival during HFD feeding and treatment with GDNP vs PBS, compared to control lean animals. One-way ANOVA with the Bonferroni correction for multiple comparisons was used to calculate statistical significance. (*p* value *<0.05; **<0.01; ***<0.001)

Systemic chronic inflammation is a hallmark of obesity. GDNP treatment inhibited the HFD-induced increase in pro-inflammatory cytokines including IL-1β, TNF-α and IL-6, and induced the anti-inflammatory cytokine, IL-10 (Figure 6A & Table S5) in plasma. Furthermore, we evaluated the inflammatory cytokine levels in adipose and liver tissues. Results suggested that GDNP treatment inhibited the expression of inflammatory cytokines induced in HFD-fed mice (Figure S6A & B). High blood sugar can lead to dehydration, dry skin, and inflammation. Indeed, a cytokine array of skin tissue from GDNP-treated HFD-fed mice revealed that the levels of skin inflammatory cytokines, including IL-33, were significantly downregulated when compared to PBS treated mice (Figure 6B-C). This reduction of inflammatory cytokines is consistent with decreased immune cell (F4/80 and CD3) infiltration in GDNP treated HFD-fed mice relative to PBS treated mice (Figure S7A & B, respectively). Recently, IL-33 was identified as an inflammatory agent in skin, specifically in dermatitis 39. GDNP treatment also prevented skin lesions and reduced the appearance of gray fur (Figure 6D). This low-grade, chronic inflammatory condition is a universal feature of aging and plays a significant role in morbidity and mortality in elderly individuals 40. We also found that GDNP-treated HFD-fed mice had a 3 - 5-month increase in life span compared to PBS-treated HFD-fed mice (Figure 6E). Collectively, these findings suggested that GDNP added to drinking water improved the overall health of HFD-fed mice throughout their life span.

## Discussion

A HFD is known to change cellular physiology and lead to the development of detrimental health outcomes such as obesity or T2D. Studies on mice and humans have suggested that chronic consumption of a HFD causes inactivation of the transcription factor Foxa2 41. Studies have further suggested that activated Foxa2 promotes insulin signaling. However, it is unclear whether diet-derived factors regulate the expression of Foxa2.

Here, for the first time, we demonstrated that ginger-derived nanoparticles (GDNP) can prevent insulin resistance by restoring homeostasis in gut epithelial and hepatic Foxa2 signaling in mice fed the HFD. GDNP treatment restored the expression of Foxa2 that was disrupted by the HFD by preventing Akt-1 mediated phosphorylation of Foxa2.

In a healthy, varied diet, multiple particles of variable size and composition are ingested, each of which could have a distinct effect on regulating Foxa2 activity. These findings provide a foundation for future studies to determine the molecular pathway that monitors the level of intracellular Foxa2 by nanoparticles from varied diets. The finding that the insulin response is regulated by diet-derived factors inducing Foxa2 may have broader implications since Foxa2 is a pioneer transcription factor that has been found to play important roles in multiple stages of mammalian life, beginning with early development, continuing during organogenesis 42, and finally in regulating metabolism and glucose homeostasis 6 43 in adults.

Foxa2 can bind nucleosomal DNA at naïve and unprogrammed chromatin regions 44 where they generate and maintain a chromatin configuration suitable for the access of other transcription factors 45, 46 such as the androgen (AR) and the estrogen receptors (ER) 47, 48. In addition, in spite of the ability of Foxa2 to bind nucleosomal DNA 46, the genomic distribution of Foxa2 is context‐ and cell type‐specific 49, 50 even in conditions of ectopic expression 51. Therefore, other transcription factors may be induced by GDNP as well and Foxa2 relies on cooperative interactions with partner transcription factors (TFs) for recruitment to DNA and induced expression of genes which may also contribute to regulating insulin signaling pathway. Although the evidence presented in this study suggests that modulation of Foxa2 in intestinal epithelial cells has therapeutic effects on high-fat diet induced obesity and diabetes, cell type specific Foxa2 genomic distribution profiles, transcriptional outcomes, and biological functions need to be further investigated in the future studies.

Besides the expression of Foxa2, the expression of other genes as indicated in Figure 1B are also affected by GDNP treatment. Studies on mice and humans have suggested that chronic consumption of a HFD causes a dysregulation of the expression of the genes involved in the insulin signaling pathway including Akt-1/2 52, IRS-1/2 53, AhR 20, 54, ARNTL 55, FGF15 56 and transcription factor Foxa2 41. In this study, we show that GDNP PA is involved in prevention of Foxa2 downregulation and that GDNP may potentially regulate the expression of other genes listed in Fig. 1b such as Akt-1/2, IRS-1/2, AhR, ARNTL, and FGF15. This finding provides the rationale for further clarifying which GDNP factor(s) plays a role in altering the expression of these genes that contribute to regulating the insulin signaling pathway.

In this study, our *in vitro* data suggest that GDNP treatment leads to reduction of pAkt-1 but not pAkt-2. Our *in vivo* results demonstrate that GDNP treatment prevents inactivation of Foxa2, and this result is associated with the reduction of the phosphorylation of Akt-1 but not Akt-2 in IECs. Previous studies have shown that phosphorylation of Foxa2 is mediated by pAkt-1 6, There are three Akt isoforms including Akt1, Akt2, and Akt3, all of which are ubiquitously expressed in the tissues. Akt1 knockout mice do not have insulin resistance 33. Akt2 knockout mice have impaired insulin action on the liver 34. Thus, *in vivo*, whether other host factor(s) is/are required for Akt-1 mediated inactivation of Foxa2 remains unclear. This issue can be further investigated by studying an animal model that has Akt selectively deleted in intestinal epithelial cells or hepatocytes. So, our findings provide a rational for further studying whether GDNP treatment leads to prevention of additional factors that inhibit activation of Foxa2.

Caution must be exercised when drawing any conclusions regarding the role of lipid PA in the context of GDNP mediated prevention of HFD induced obesity and insulin resistance. GDNP PA can prevent phosphorylation of Foxa2 by inhibiting Akt-1 activation, however, it is unlikely that GDNP PA is fully responsible for prevention of HFD induced obesity and insulin resistance since more than one factor is dysregulated and contributes to the development of this pathological phenotype. Because GDNP is composed of multiple factors, it could potentially correct multiple dysregulated pathways during the development of obesity and insulin resistance.

In summary, our findings support further exploration of the development of edible nanoparticle-based strategies for the prevention and treatment of metabolic disease. In addition, this strategy would likely have few side effects, because the system is based on edible plant carriers to deliver therapeutic agents to the intestinal epithelial cells.

## Figures and Tables

**Table 1:** Detailed description of high fat diet used in the study

Class description	Ingredient	Grams
Protein	Casein, Lactic, 30Mesh	200
Protein	Cystine, L	3
Carbohydrate	Lodex 10,	125
Carbohydrate	Fine granulated Sucrose	72.8
Fiber	Solka Floc, FCC200	50
Fat	Lard	245
Fat	Soybean oil, USP	25
Mineral	S10026B	50
Vitamin	Choline bitartrate	2
Vitamin	V10001C	1
Dye	Dye Blue FD&C #1, Alum. Lake 35-42%	0.05
