# Supplementary material for: Ginger nanoparticles mediated induction of Foxa2 prevents high-fat diet-induced insulin resistance
Source: Theranostics. 2022 Jan 1;12(3):1388–403. doi: 10.7150/thno.62514 (PMC8771553; doi:10.7150/thno.62514)
Supplement: Supplementary file 1 — Supplementary figures and tables. [file thnov12p1388s1.pdf]

Supplementary information

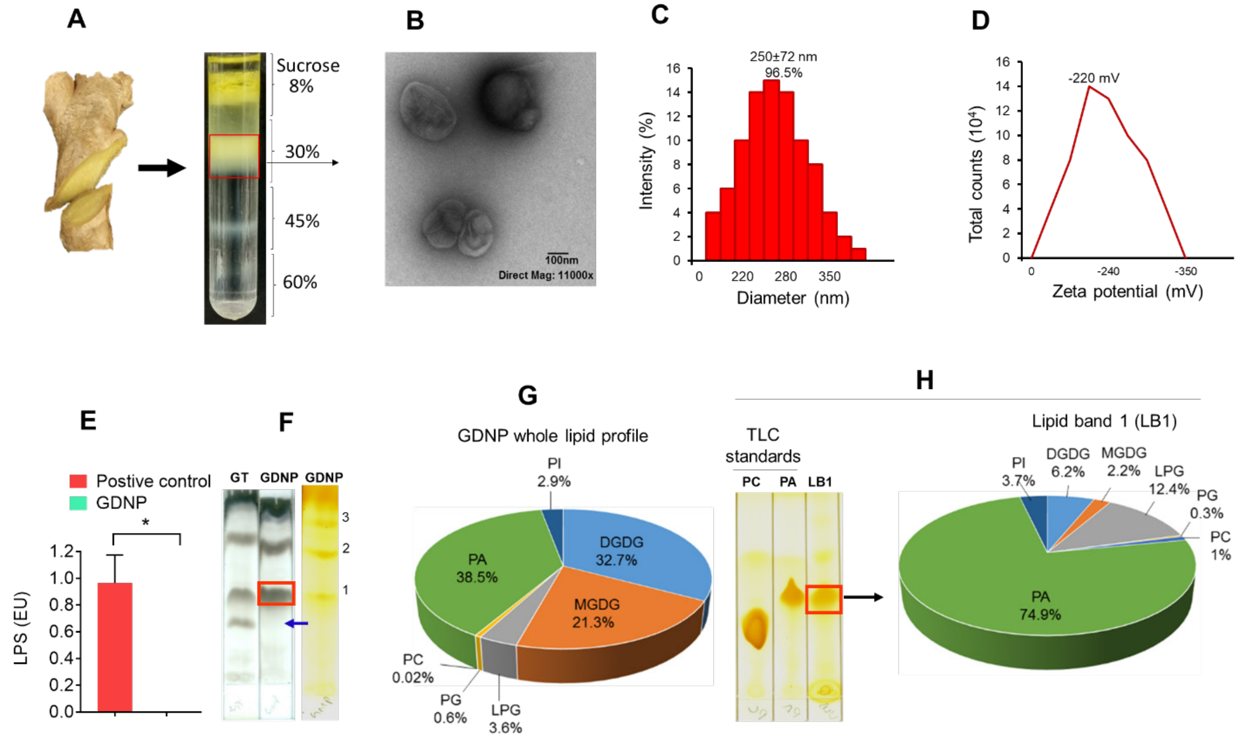

Figure S1: Characterization of ginger nanoparticles (GDNP).

- **A.** Depiction of the sucrose gradient purification of ginger-derived nanoparticles (GDNP).
- **B.** Electron micrograph of purified GDNP from the red box in panel A.
- **C.** GDNP size distribution as determined using the Zeta sizer.
- **D.** Zeta potential of GDNP as determined using the Zeta sizer.
- **E.** LPS detection in GDNP. Student *t* (one-tailed) test. *p* value *<0.05.
- **F.** Thin layer chromatography (TLC) profile of lipids derived from whole ginger root (GT) and ginger nanoparticles (GDNP) stained with CuSO4 or iodine fumes. The red box shows lipid band 1, which is responsible for induction of Foxa2. The blue arrow indicates the absent of the lipid band in GDNP.
- **G.** Pie chart representing the lipid composition of GDNP analyzed by quadrupole mass spectrometry (Q-MS).
- **H.** TLC lipid profile of lipid band 1 (LB1). PA and PC were used as standards in the TLC analysis.
- **I.** GDNP dose per day in drinking water.

Student *t* test (one tailed) was used to calculate statistical significance. (*p* value *<0.05).

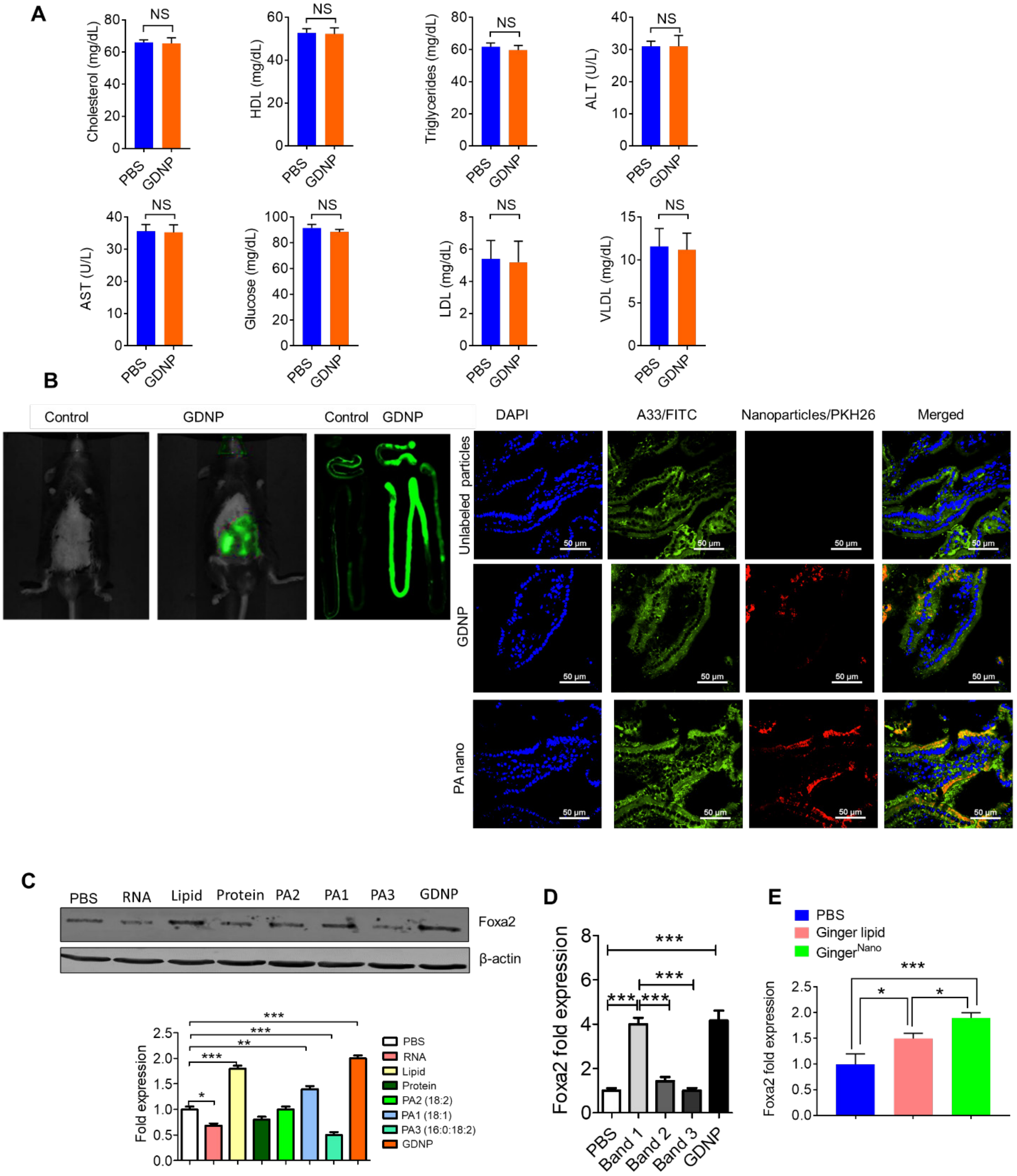

A
Cholesterol (mg/dL)
HDL (mg/dL)
Triglycerides (mg/dL)
ALT (U/L)
AST (U/L)
Glucose (mg/dL)
LDL (mg/dL)
VLDL (mg/dL)
NS
PBS
GDNP
B
Control
GDNP
Control GDNP
DAPI
A33/FITC
Nanoparticles/PKH26
Merged
Unlabeled particles
GDNP
PA nano
50 μm
C
PBS RNA Lipid Protein PA2 PA1 PA3 GDNP
Foxa2
β-actin
Fold expression
PBS
RNA
Lipid
Protein
PA2 (18:2)
PA1 (18:1)
PA3 (16:0:18:2)
GDNP
D
Foxa2 fold expression
PBS
Band 1
Band 2
Band 3
GDNP
E
PBS
Ginger lipid
Ginger^Nano
Foxa2 fold expression

Figure S2: Gut epithelial cell uptake of GDNP.

- **A.** Lipid profile of plasma derived from lean mice treated with PBS or GDNP after 2 weeks (n = 5 per group).
- **B.** Live imaging of mouse gavaged the DIR labeled GDNP after 6 hours (GDNP panel). Small and large intestine showing the GDNP or PA nanoparticle uptake and A33 positive cells uptake of PKH26 labeled GDNP/PA nanoparticles. Control represented as unlabeled GDNP.
- **C.** Foxa2 protein expression in MC-38 cells following treatment with PBS, GDNP RNA, protein extracts with phosphatidic acids (PAs) lipids or GDNP.
- **D.** Foxa2 mRNA expression following treatment with either PBS, each band of GDNP lipids, or complete GDNP.
- **E.** QRT PCR for Foxa2 mRNA expression in MC-38 cells treated with ginger lipid (linear form) and nanoparticles.

One-way ANOVA with a Bonferroni correction for multiple comparisons was used to calculate statistical significance. (*p* value *<0.05; **<0.01; ***<0.001).

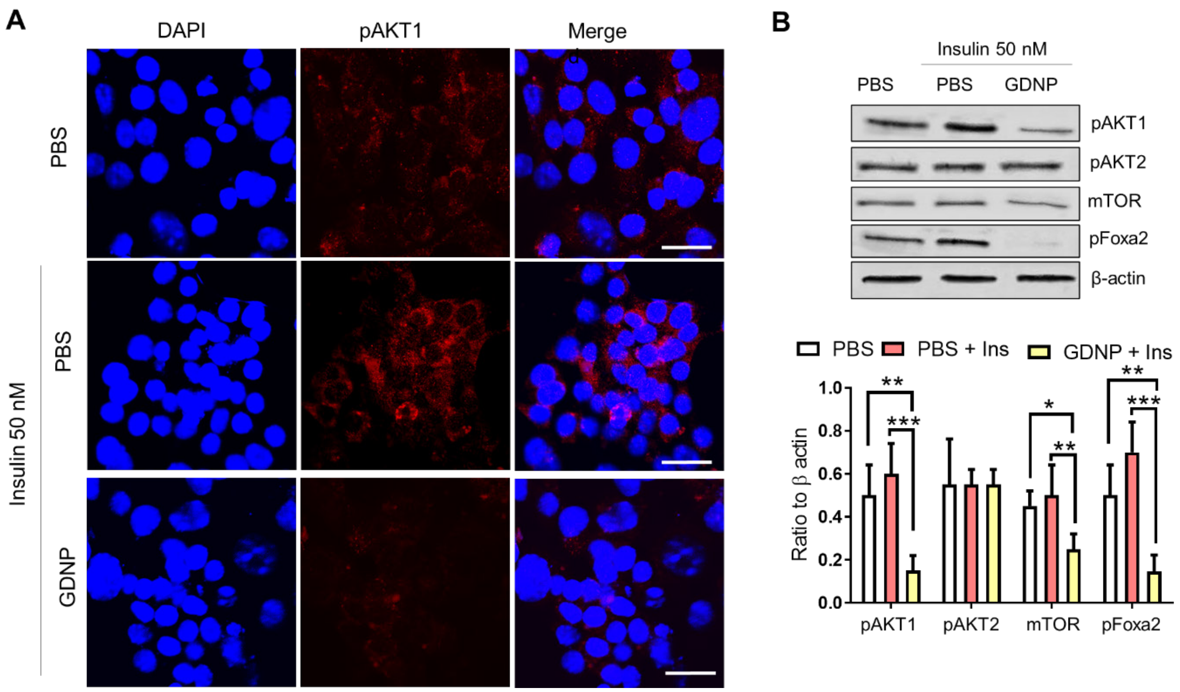

Figure S3: GDNP treatment inhibits the pAKT-1-mediated phosphorylation of Foxa2.

- **A.** Confocal images showing expression of pAKT1 (red) in MC-38 cells cultured with 50 nM insulin and PBS or GDNP. Scale bar is 20 µM.
- **B.** Western blots of pAKT1, pAKT2 mTOR and pFoxa2 expression in MC-38 cells cultured with 50 nM insulin and treated with PBS or GDNP.

One-way ANOVA with a Bonferroni correction for multiple comparisons was used to calculate statistical significance. (*p* value *<0.05; **<0.01; ***<0.001).

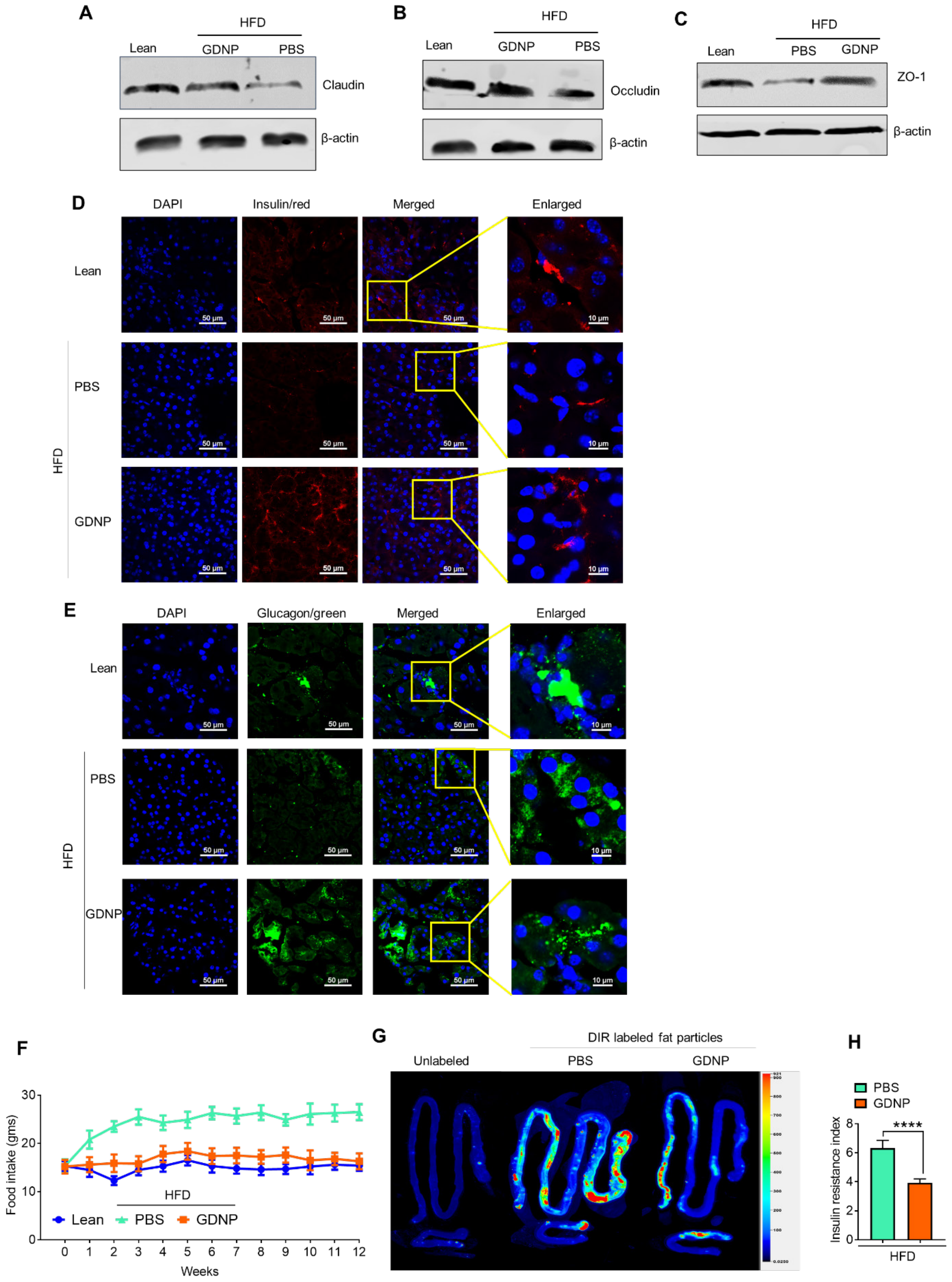
A
HFD
Lean
GDNP
PBS
Claudin
β-actin
B
HFD
Lean
GDNP
PBS
Occludin
β-actin
C
HFD
Lean
PBS
GDNP
ZO-1
β-actin
D
DAPI
Insulin/red
Merged
Enlarged
Lean
PBS
GDNP
HFD
50 µm
10 µm
E
DAPI
Glucagon/green
Merged
Enlarged
Lean
PBS
GDNP
HFD
50 µm
10 µm
F
Food intake (gms)
Weeks
HFD
Lean
PBS
GDNP
G
DIR labeled fat particles
Unlabeled
PBS
GDNP
H
PBS
GDNP
Insulin resistance index
****
HFD

Figure S4: GDNP enhances the gut barrier integrity in HFD mice.

- **A. – C.** Western blots for tight junction proteins Claudin (A), Occludin (B) and ZO-1 (C) for small intestinal tissue.
- **D.** Confocal images depicting the expression of insulin in pancreatic tissues derived from lean and HFD mice treated with PBS or GDNP in fed condition (n = 5 per group).
- **E.** Confocal images depicting the expression of glucagon in pancreatic tissues derived from lean and HFD-fed mice treated with PBS or GDNP in fed condition (n = 5 per group).
- **F.** Food intake for lean and HFD-fed mice treated with PBS and GDNP.
- **G.** Images from small and large intestine for DIR labeled fat particles uptake.
- **H.** Insulin resistance index for HFD-fed mice treated with PBS or GDNP.

Student *t* test (one tailed) was used to calculate statistical significance. (*p* value ****<0.0001).

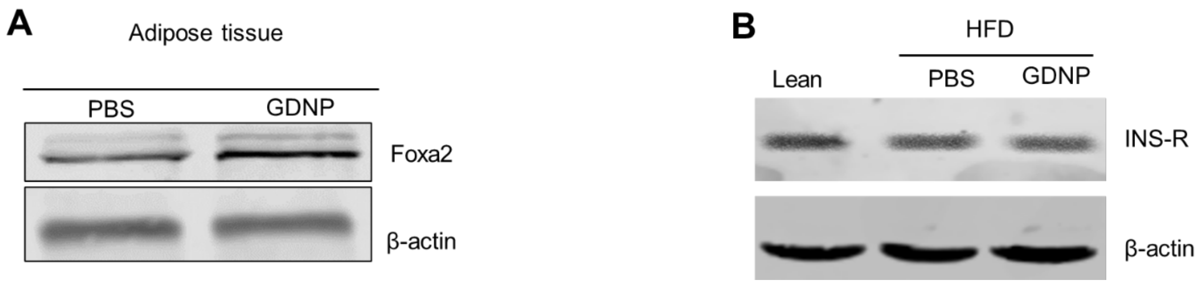

Figure S5: Western blots for Foxa2 (A) and Insulin receptor (B) in adipose tissues.

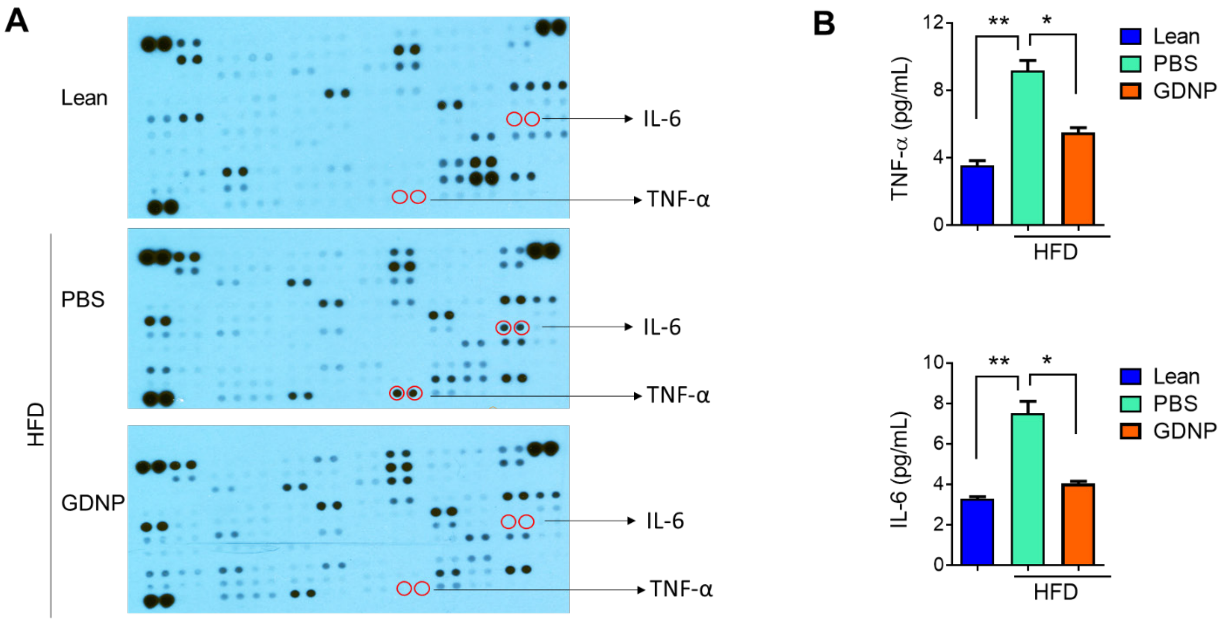

Figure S6: GDNP treatment prevent the mice from chronic inflammation

A. Cytokine array of adipose tissue lysates derived from lean and HFD-fed mice treated with PBS or GDNP (n = 5 per group).

B. Levels of inflammatory cytokines TNF-α and IL-6 in liver tissue lysates determined by ELISA (n = 5 per group).

One-way ANOVA with a Bonferroni correction for multiple comparisons was used to calculate statistical significance. (*p* value *<0.05; **<0.01).

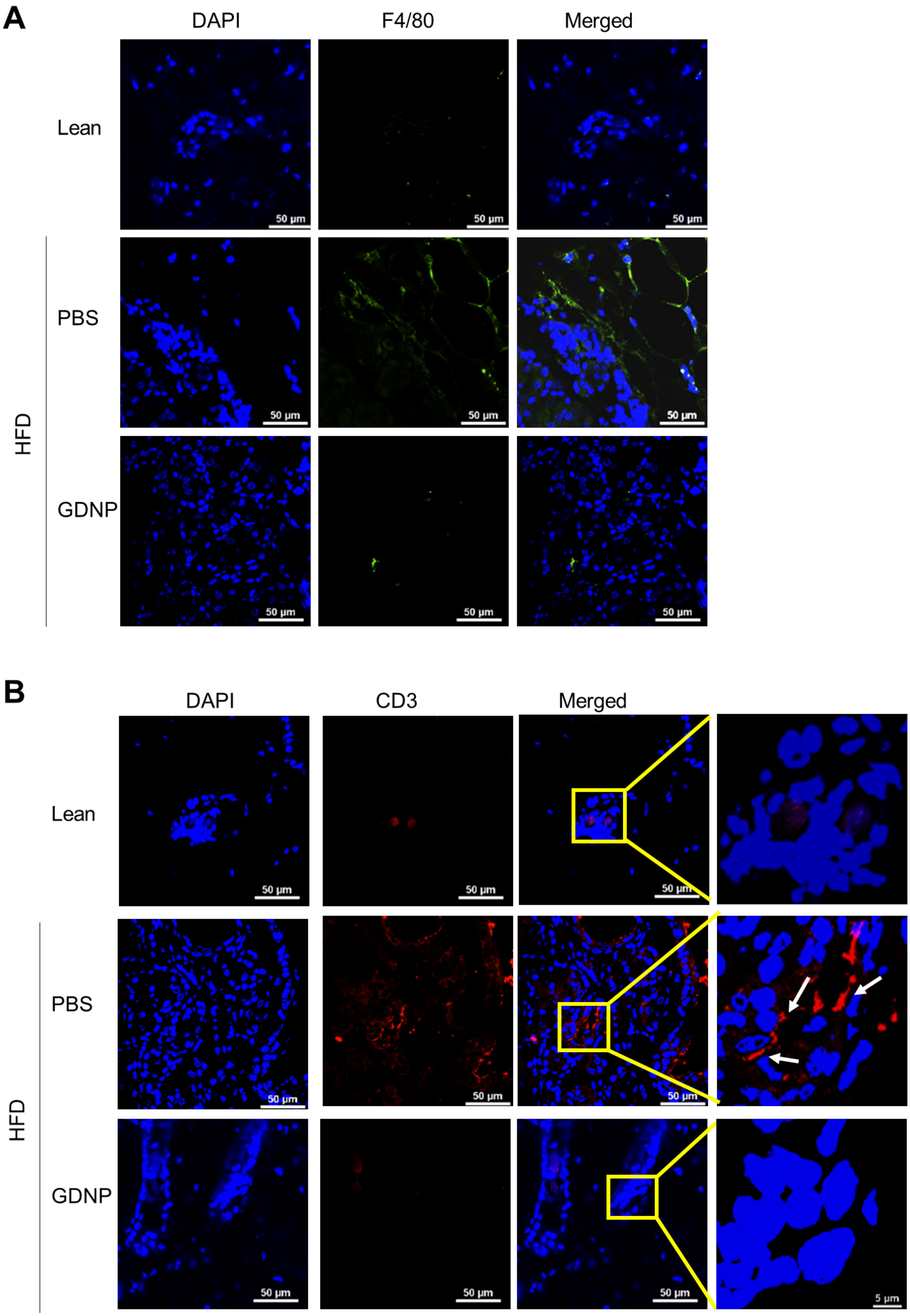

A
DAPI
F4/80
Merged
Lean
HFD
PBS
GDNP
50 µm
B
DAPI
CD3
Merged
Lean
HFD
PBS
GDNP
50 µm
5 µm

Figure S7: GDNP inhibit the immune cells infiltration caused by long-term HFD in skin tissues.

A. –**B.** Visualization of macrophage ($F4/80^+$; a) and T cell ($CD3^+$; b) infiltration (white arrow) in HFD-fed mouse skin tissues by confocal microscopy.

Table S1: Primers used in the study

Target	Sequence (5' – 3')	Tm
Foxa2F	CCCTACGCCAACATGAACTCG	64.5
Foxa2R	GTTCTGCCGGTAGAAAGGGA	62.4

Table S2: List of antibodies used in the study

S. No.	Target	Application	Source	Cat. No.
1	Foxa2	IF/Western	R&D Systems	MAB2400
2	pFoxa2	IF/WB	Thermo Scientific	710680
3	AHR	WB	Santa Cruz	Sc133088
4	A33	IF/Flow	Biorybt	Orb15687
5	pAkt-1	IF/WB	Cell Signaling	9018S
6	pAkt-2	IF/WB	Cell Signaling	8599S
7	B-Actin	WB	Santa Cruz	Sc47778
8	GAPDH	WB	Santa Cruz	Sc47724
9	F4/80	IF	eBioscience	14-4801-82

Table S3: Quadrupole MS analysis of GDNP whole lipids and lipid band 1 (nm).

**Mass**	**Compound Formula**	**Compound Name**	**GDNP Whole lipid (nm)**	**Lipid band 1 (nm)**
926.6	C49H80O15	DGDG(34:6)	0.00	0.00
928.6	C49H82O15	DGDG(34:5)	0.00	0.02
930.6	C49H84O15	DGDG(34:4)	0.00	0.00
932.6	C49H86O15	DGDG(34:3)	0.42	0.21
934.6	C49H88O15	DGDG(34:2)	2.86	1.03
936.6	C49H90O15	DGDG(34:1)	5.13	0.98
954.6	C51H84O15	DGDG(36:6)	0.51	0.00
956.6	C51H86O15	DGDG(36:5)	2.01	0.24
958.6	C51H88O15	DGDG(36:4)	9.61	1.21
960.6	C51H90O15	DGDG(36:3)	6.69	1.24
962.6	C51H92O15	DGDG(36:2)	3.56	0.00
964.7	C51H94O15	DGDG(36:1)	1.88	1.22
982.6	C53H88O15	DGDG(38:6)	0.00	0.00
984.6	C53H90O15	DGDG(38:5)	0.00	0.00
986.6	C53H92O15	DGDG(38:4)	0.00	0.00
988.7	C53H94O15	DGDG(38:3)	0.00	0.00
**Total DGDG**		**Total DGDG**	**32.66**	**6.17**
764.5	C43H70O10	MGDG(34:6)	0.00	0.17
766.5	C43H72O10	MGDG(34:5)	0.09	0.00

768.5	C43H74O10	MGDG(34:4)	0.03	0.05
770.5	C43H76O10	MGDG(34:3)	2.34	0.10
772.6	C43H78O10	MGDG(34:2)	0.18	0.00
774.6	C43H80O10	MGDG(34:1)	1.20	0.00
792.5	C45H74O10	MGDG(36:6)	1.98	0.05
794.5	C45H76O10	MGDG(36:5)	4.00	0.07
796.6	C45H78O10	MGDG(36:4)	7.33	0.75
798.6	C45H80O10	MGDG(36:3)	2.83	0.16
800.6	C45H82O10	MGDG(36:2)	0.94	0.00
802.6	C45H84O10	MGDG(36:1)	0.00	0.00
820.6	C47H78O10	MGDG(38:6)	0.00	0.12
822.6	C47H80O10	MGDG(38:5)	0.33	0.00
824.6	C47H82O10	MGDG(38:4)	0.00	0.00
826.6	C47H84O10	MGDG(38:3)	0.00	0.76
**Total MGDG**		**Total MGDG**	**21.25**	**2.23**
738.5	C38H73O10P	PG(32:1)	0.05	0.02
740.5	C38H75O10P	PG(32:0)	0.04	0.13
760.5	C40H71O10P	PG(34:4)	0.03	0.00
762.5	C40H73O10P	PG(34:3)	0.06	0.00
764.5	C40H75O10P	PG(34:2)	0.18	0.04
766.5	C40H77O10P	PG(34:1)	0.02	0.07
768.5	C40H79O10P	PG(34:0)	0.05	0.00
784.5	C42H71O10P	PG(36:6)	0.01	0.00
786.5	C42H73O10P	PG(36:5)	0.02	0.00
788.5	C42H75O10P	PG(36:4)	0.01	0.00
790.5	C42H77O10P	PG(36:3)	0.03	0.02
792.5	C42H79O10P	PG(36:2)	0.04	0.00
794.6	C42H81O10P	PG(36:1)	0.02	0.03
**Total PG**		**Total PG**	**0.56**	**0.32**
500.3	C22H43O9P	LPG(16:1)	0.72	3.72
502.3	C22H45O9P	LPG(16:0)	0.72	0.00
524.3	C24H43O9P	LPG(18:3)	0.68	3.16
526.3	C24H45O9P	LPG(18:2)	1.06	1.92
528.3	C24H47O9P	LPG(18:1)	0.40	3.59
**Total LysoPG**		**Total LysoPG**	**3.58**	**12.39**
494.3	C24H48O7PN	LPC(16:1)	0.00	0.00
496.3	C24H50O7PN	LPC(16:0)	0.00	0.53
518.3	C26H48O7PN	LPC(18:3)	0.00	0.00
520.3	C26H50O7PN	LPC(18:2)	0.01	0.00
522.3	C26H52O7PN	LPC(18:1)	0.01	0.53

524.4	C26H54O7PN	LPC(18:0)	0.00	0.00
**Total LysoPC**		**Total LysoPC**	**0.02**	**1.11**
452.3	C21H42O7PN	LPE(16:1)	0.03	0.00
454.3	C21H44O7PN	LPE(16:0)	0.01	0.00
476.3	C23H42O7PN	LPE(18:3)	0.00	0.00
478.3	C23H44O7PN	LPE(18:2)	0.02	0.00
480.3	C23H46O7PN	LPE(18:1)	0.01	0.00
**Total LysoPE**		**Total LysoPE**	**0.07**	**0.00**
734.6	C40H80O8PN	PC(32:0)	0.00	0.01
754.5	C42H76O8PN	PC(34:4)	0.00	0.00
756.5	C42H78O8PN	PC(34:3)	0.01	0.00
758.6	C42H80O8PN	PC(34:2)	0.03	0.00
760.6	C42H82O8PN	PC(34:1)	0.00	0.00
778.5	C44H76O8PN	PC(36:6)	0.00	0.00
780.5	C44H78O8PN	PC(36:5)	0.00	0.00
782.6	C44H80O8PN	PC(36:4)	0.00	0.00
784.6	C44H82O8PN	PC(36:3)	0.01	0.00
786.6	C44H84O8PN	PC(36:2)	0.01	0.00
788.6	C44H86O8PN	PC(36:1)	0.00	0.00
806.6	C46H80O8PN	PC(38:6)	0.00	0.00
808.6	C46H82O8PN	PC(38:5)	0.00	0.00
810.6	C46H84O8PN	PC(38:4)	0.00	0.00
812.6	C46H86O8PN	PC(38:3)	0.00	0.02
814.6	C46H88O8PN	PC(38:2)	0.00	0.00
836.6	C48H86O8PN	PC(40:5)	0.00	0.00
838.6	C48H88O8PN	PC(40:4)	0.00	0.00
840.6	C48H90O8PN	PC(40:3)	0.00	0.00
842.7	C48H92O8PN	PC(40:2)	0.00	0.00
**Total PC**		**Total PC**	**0.06**	**0.03**
686.5	C37H68O8PN	PE(32:3)	0.00	0.00
688.5	C37H70O8PN	PE(32:2)	0.03	0.00
690.5	C37H72O8PN	PE(32:1)	0.07	0.15
692.5	C37H74O8PN	PE(32:0)	0.01	0.00
712.5	C39H70O8PN	PE(34:4)	0.00	0.00
714.5	C39H72O8PN	PE(34:3)	0.02	0.00
716.5	C39H74O8PN	PE(34:2)	0.16	0.00
718.5	C39H76O8PN	PE(34:1)	0.01	0.00
736.5	C41H70O8PN	PE(36:6)	0.00	0.00
738.5	C41H72O8PN	PE(36:5)	0.01	0.00
740.5	C41H74O8PN	PE(36:4)	0.00	0.00

742.5	C41H76O8PN	PE(36:3)	0.01	0.01
744.5	C41H78O8PN	PE(36:2)	0.01	0.00
746.6	C41H80O8PN	PE(36:1)	0.00	0.00
764.5	C43H74O8PN	PE(38:6)	0.00	0.00
766.5	C43H76O8PN	PE(38:5)	0.00	0.00
768.5	C43H78O8PN	PE(38:4)	0.00	0.00
770.6	C43H80O8PN	PE(38:3)	0.00	0.00
798.6	C45H84O8PN	PE(40:3)	0.00	0.00
800.6	C45H86O8PN	PE(40:2)	0.00	0.00
824.6	C47H86O8PN	PE(42:4)	0.00	0.00
826.6	C47H88O8PN	PE(42:3)	0.00	0.00
828.6	C47H90O8PN	PE(42:2)	0.00	0.00
**Total PE**		**Total PE**	**0.33**	**0.16**
822.5	C41H73O13P	PI(32:3)	0.11	0.13
824.5	C41H75O13P	PI(32:2)	0.08	0.00
826.5	C41H77O13P	PI(32:1)	0.08	0.21
828.5	C41H79O13P	PI(32:0)	0.12	0.00
848.5	C43H75O13P	PI(34:4)	0.03	0.00
850.5	C43H77O13P	PI(34:3)	0.20	0.33
852.5	C43H79O13P	PI(34:2)	0.80	1.67
854.5	C43H81O13P	PI(34:1)	0.21	0.87
872.5	C45H75O13P	PI(36:6)	0.06	0.05
874.5	C45H77O13P	PI(36:5)	0.19	0.00
876.5	C45H79O13P	PI(36:4)	0.73	0.19
878.5	C45H81O13P	PI(36:3)	0.24	0.15
880.6	C45H83O13P	PI(36:2)	0.02	0.01
882.6	C45H85O13P	PI(36:1)	0.00	0.00
**Total PI**		**Total PI**	**2.87**	**3.62**
756.5	C40H70O10PN	PS(34:4)	0.00	0.00
758.5	C40H72O10PN	PS(34:3)	0.00	0.00
760.5	C40H74O10PN	PS(34:2)	0.02	0.00
762.5	C40H76O10PN	PS(34:1)	0.00	0.00
780.5	C42H70O10PN	PS(36:6)	0.00	0.00
782.5	C42H72O10PN	PS(36:5)	0.01	0.00
784.5	C42H74O10PN	PS(36:4)	0.01	0.01
786.5	C42H76O10PN	PS(36:3)	0.00	0.00
788.5	C42H78O10PN	PS(36:2)	0.00	0.00
790.6	C42H80O10PN	PS(36:1)	0.00	0.01
808.5	C44H74O10PN	PS(38:6)	0.00	0.00
810.5	C44H76O10PN	PS(38:5)	0.00	0.00

812.5	$C_{44}H_{78}O_{10}PN$	PS(38:4)	0.00	0.00
814.6	$C_{44}H_{80}O_{10}PN$	PS(38:3)	0.00	0.00
816.6	$C_{44}H_{82}O_{10}PN$	PS(38:2)	0.00	0.01
818.6	$C_{44}H_{84}O_{10}PN$	PS(38:1)	0.00	0.00
840.6	$C_{46}H_{82}O_{10}PN$	PS(40:4)	0.00	0.00
842.6	$C_{46}H_{84}O_{10}PN$	PS(40:3)	0.00	0.00
844.6	$C_{46}H_{86}O_{10}PN$	PS(40:2)	0.00	0.00
846.6	$C_{46}H_{88}O_{10}PN$	PS(40:1)	0.00	0.00
868.6	$C_{48}H_{86}O_{10}PN$	PS(42:4)	0.00	0.00
870.6	$C_{48}H_{88}O_{10}PN$	PS(42:3)	0.00	0.00
872.6	$C_{48}H_{90}O_{10}PN$	PS(42:2)	0.04	0.00
874.6	$C_{48}H_{92}O_{10}PN$	PS(42:1)	0.01	0.00
898.6	$C_{50}H_{92}O_{10}PN$	PS(44:3)	0.00	0.00
900.7	$C_{50}H_{94}O_{10}PN$	PS(44:2)	0.01	0.00
**Total PS**		**Total PS**	**0.12**	**0.04**
666.5	$C_{35}H_{69}O_8P$	PA(32:0)	0.53	1.60
682.4	$C_{37}H_{61}O_8P$	PA(34:6)	0.00	0.00
684.4	$C_{37}H_{63}O_8P$	PA(34:5)	0.00	0.00
686.4	$C_{37}H_{65}O_8P$	PA(34:4)	0.03	0.00
688.5	$C_{37}H_{67}O_8P$	PA(34:3)	1.10	2.12
690.5	$C_{37}H_{69}O_8P$	PA(34:2)	16.31	32.32
692.5	$C_{37}H_{71}O_8P$	PA(34:1)	3.41	8.02
710.4	$C_{39}H_{65}O_8P$	PA(36:6)	0.02	0.00
712.5	$C_{39}H_{67}O_8P$	PA(36:5)	0.94	1.83
714.5	$C_{39}H_{69}O_8P$	PA(36:4)	9.40	16.79
716.5	$C_{39}H_{71}O_8P$	PA(36:3)	5.02	8.61
718.5	$C_{39}H_{73}O_8P$	PA(36:2)	1.71	3.66
**Total PA**		**Total PA**	**38.47**	**74.94**

Table S4: Log2 values for the expression of genes in the Affymetrix array of small intestinal tissues of HFD-fed mice treated with PBS or GDNP.

	PBS1	PBS2	PBS3	GDNP1	GDNP2	GDNP3
Foxa2	0.245093	0.269274	0.274455	0.281342	0.280604	0.277839
Vamp7	0.551879	0.568156	0.572387	0.674514	0.642258	0.595921
AhR	0.410544	0.402235	0.437675	0.386698	0.356065	0.339581
AhRR	0.292204	0.318994	0.307434	0.351972	0.284516	0.287762
ME1	0.595625	0.427374	0.430967	0.411418	0.389044	0.347299
AKT1	0.659002	0.611732	0.569033	0.652737	0.643376	0.564498
AKT2	0.421761	0.417318	0.424259	0.420247	0.409167	0.404079

Akt1Sub	0.596186	0.575419	0.59251	0.562684	0.538849	0.517089
ARNTL	0.549635	0.577095	0.622694	0.530312	0.502515	0.496692
Cyp7a1	0.249019	0.265363	0.256568	0.247793	0.231414	0.235943
Cyp8b1	0.226584	0.257542	0.223589	0.247204	0.227501	0.23043
NR1H4	0.63152	0.707821	0.699273	0.781636	0.717719	0.70011
Rptor	0.319686	0.353631	0.350475	0.388464	0.374511	0.379824
Deptor	0.46046	0.575978	0.593069	0.532666	0.5109	0.573319
mTor	0.430174	0.47933	0.427613	0.393172	0.481274	0.46527
FGF15	0.771172	0.66257	0.680268	0.843437	0.805478	0.76957

Table S5: Plasma cytokines or growth factors expressed by HFD-fed mice treatment with PBS or GDNP.

Name of cytokine/factor	Fold change GDNP vs PBS	Name of cytokine/factor	Fold change GDNP vs PBS
EGF	13.31	WISP-1	-2.63
ANGPT-1	6.82	IL-6	-2.22
CCL5	2.42	IL-33	-2.01
Leptin	2.00	TSG-14	-1.97
FGF-21	1.88	CXCL13	-1.96
CXCL10	1.87	CXCL1	-1.95
PDGF-BB	1.72	VEGF	-1.91
CCL11	1.71	Serpin E1	-1.86
IL-1 α	1.69	CD14	-1.81
HGF	1.68	IL-27p28	-1.73
IL-3	1.62	Pentraxin	-1.66
CCL12	1.47	G-CSF	-1.64
DKK-1	1.41	MMP-3	-1.62
FGF-1	1.38	MPO	-1.59
IL-10	1.36	Pref-1	-1.58
Areg	1.34	IL-28	-1.57
IL-11	1.32	C1q R1	-1.55
CCL17	1.24	CCL2	-1.49
		POSTN	-1.49
		CX3CL1	-1.49
		CXCL16	-1.48
		IL-15	-1.45
		IGFBP-1	-1.44

		IL-7	-1.44
		RAGE	-1.40
		IFN-γ	-1.39
		TIM-1	-1.39
		Proliferin	-1.34
		END	-1.33
		LCN-2	-1.33
		ANGPT-2	-1.28
		LIF	-1.28
		BAFF	-1.25
		TNF-α	-1.25
